# Verification study on how macrofungal fruitbody formation can be predicted by artificial neural network

**DOI:** 10.1038/s41598-023-50638-8

**Published:** 2024-01-02

**Authors:** Katalin Somfalvi-Tóth, Ildikó Jócsák, Ferenc Pál-Fám

**Affiliations:** Department of Agronomy, Institute of Agronomy, Hungarian University of Agriculture and Life Sciences, 40 Guba S. Str., Kaposvár, 7400 Hungary

**Keywords:** Ecology, Plant sciences, Ecology, Environmental sciences

## Abstract

The occurrence and regularity of macrofungal fruitbody formation are influenced by meteorological conditions; however, there is a scarcity of data about the use of machine-learning techniques to estimate their occurrence based on meteorological indicators. Therefore, we employed an artificial neural network (ANN) to forecast fruitbody occurrence in mycorrhizal species of *Russula* and *Amanita*, utilizing meteorological factors and validating the accuracy of the forecast of fruitbody formation. Fungal data were collected from two locations in Western Hungary between 2015 and 2020. The ANN was the commonly used algorithm for classification problems: feed-forward multilayer perceptrons with a backpropagation algorithm to estimate the binary (Yes/No) classification of fruitbody appearance in natural and undisturbed forests. The verification indices resulted in two outcomes: however, development is most often studied by genus level, we established a more successful, new model per species. Furthermore, the algorithm is able to successfully estimate fruitbody formations with medium to high accuracy (60–80%). Therefore, this work was the first to reliably utilise the ANN approach of estimating fruitbody occurrence based on meteorological parameters of mycorrhizal specified with an extended vegetation period. These findings can assist in field mycological investigations that utilize sporocarp occurrences to ascertain species abundance.

## Introduction

Actual weather conditions play an important role in driving the fruiting pattern of macrofungal species. Current studies have shown that the formation of fruitbodies is determined by several environmental factors such as light, air temperature, physical damage, water and nutrient availability and, among others, hormonal effects^1^. Other influencing factors are the physiology and spatial distribution of the host or mycorrhizal partner, as well as the competition for resources. Water availability and air temperature, or more precisely, their combination are among the most influencing environmental factors for determining the occurrence of fruitbody in natural fungal communities. In a Europe-wide survey of several different habitats, including Austria, Norway, Switzerland and the United Kingdom, it was possible to demonstrate a correlation between total fruitbody production and certain climatic conditions, such as temperature and/or precipitation^2^. Based on the above-mentioned observations, the emergence and/or the temporal shift of fruiting body occurrences in a habitat may be an indicator of actual weather conditions and, more importantly, may reveal climate change-related processes as well^2,3^.

The fruitbody-based study of fungi is relatively easy and inexpensive, and in many cases it allows rapid, almost immediate results. In the field of conservation, fungi are more responsive and quickly indicate environmental changes than plants through the presence or absence of fruitbodies. Therefore, it is possible to detect environmental impacts, such as those caused by human activity, as early as the next fruiting body formation period, thus anticipating or preventing subsequent changes in the community of a habitat^4^. Not least among these fungi are a number of endangered species, including species important for human medicine and food. The importance of studying macrofungi was pointed out by Cao et al.^5^. The fungal fruitbody, by analogy, corresponds to that of the fruit of the angiosperm plants. This is a major difficulty when designing mycological studies based on fruitbodies^5^. They suggest that the understanding of these indicative properties of fungi is still in its infancy, therefore the methodology of investigation should include the most modern thecnologies, such as DNA (meta)barcoding, or artificial intelligence (AI).

There is strong statistical relationship among weather patterns and fruitbody formation, among which air temperature and precipitation are the most frequently studied parameters. Bonet et al.^6^ used empirical linear models to predict fungal production based on topographic characteristics (slope, total basal area of trees in a stand, elevation above sea level, etc.) as main drivers, while the weather component was entered as a correction factor into the equation to eliminate random year effect. Besides the obvious effects of temperature and rainfall Talley et al.^7^ approached the problem from another aspect: they studied fungal sporocarp production in terms of the duration of the time interval of favourable or unfavourable weather conditions for fruiting body formation. Krebs et al.^8^ applied multiple regression models to estimate the mushroom crobs based on the monthly mean temperature and the monthly sum of precipitation in the southwestern Yukon (Canada). A more sophisticated method was introduced by Taye et al.^9^. In their study, edible mushroom production was related to different meteorological and habitat conditions and soil parameters. Firstly, a logistic function was applied to determine the probability of the occurrence of fruitbody formation. In the second step of this research, using these preliminary results as conditional probabilities, the mushroom yield was estimated by means of gamma regression^9^. Laganá et al.^10^ studied the effects of microclimatic elements on the periodicity, fluctuation and succession of macrofungal species in Tuscany, Italy, using Pearson-correlation, correspondence analysis and found that the used method could not able to find linear statistical relationship between the number of fruitbody and the analysed weather parameters.

The estimation (prediction) of sporocarp occurrences based on weather parameters is challenging because it requires non-linear modeling^11^. The main limiting factor of studies based on fruitbodies is determining the exact time of fructification. Sporocarp formation is driven by the combined effects of several environmental factors. Therefore, it is advisable to target the investigations on certain fungal species, not on fungal communities or functional groups.

According to Arnolds^12^, the duration of the examination is determined by two factors: firstly, the fluctuation in the quality and quantity of the fruitbodies, because fruitbodies are not producing in certain years, and it produces with different abundance in each year. On the other hand, the frequency of the examination is determined by the fruitbodies’ periodicity and the limited time of their existence (a few hours to several weeks). Periodicity means that a particular species of fungus develops a fruitbody only at a certain time of the year, for example only in spring or only in winter. The most common species are those that grow from mid-summer to autumn^1^. Fluctuation in fruiting means that in certain years, depending on weather factors, a fungal species that is present in the area 'vegetatively' in the form of mycelium does not develop a fruitbody, whereas it does before and after a given year. In fungi, the formation of fruitbodies is a prerequisite for sexual reproduction, but, unlike in plants and animals, it does not necessarily occur every year. If the environmental conditions in a given year are not favourable, then that year will be a year of failure, with no fruiting and no sexual reproduction. However, the more or less regular occurrence of sexual reproduction is essential for the survival of the species. Periodicity and fluctuation have long been a known problem, and several attempts have been made to find appropriate methods to study biocoenosis^13^. To map fungal fruitbodies, periodicity requires that several samples be planned in a given year to find the fruitbody (Perini et al. 1996). Because of fluctuation, the fungal species pool of a given area must be surveyed over several years. Overall, surveying a mycota of macrofungi is considerably more complex work than, for example, surveying the vegetation in a given biocoenosis^14^.

There are several examples of long-term studies in the fungal scientific literature. The authors tried to solve the problem of capturing sporocarps in the field in different ways. Runge^15,16^ made long-term, multi-year studies, even longer ones than 10 years. In her 11-year study, 80% of the total number of species was recorded only in the fifth year, while in Krisai-Greilhuber's^17^ 10-year study, 93% of the total number of species was documented in the fifth year. Such long-term surveys are few in nu, and nowadays, days surveys lasting 3–5 years are more common. According to the protocol developed for the macrofungi component of the Hungarian National Biodiversity Monitoring System, a 3-year period is considered as one survey, with 5–6 samples per year^18^. Based on this protocol, fungi monitoring is continuously carried out in several forest reserves in Hungary.Others, such as Senn-Irlet^19^ and Arnolds^20^ applied several sampling plots and many field visits.

Another important feature is the mosaic-like distribution of fungi in each habitat^21^. For this reason, it has not been possible to define a standard sampling plot size (minimal area), as in the case of plants, that would allow us to infer the mycological characteristics of the whole habitat, i.e. be representative. Thus, when compiling the species composition, the whole habitat has to be surveyed in order to find the fruiting bodies of all species. For this reason, sample plot sizes have ranged from 1 m^2^ to the size of the whole habitat in the literature Sample plots between 100 and 1000 m^2^ were the most commonly used. These have been summarized in a previous work^22^. Due to the lack of representativeness, studies in fixed size plots are excellent for detecting, for example, functional groups and changes in functions, but not for assessing species richness^23,24^.

In order to resolve these issues, one possible solution may be the application of artifical neural network (ANN); first introduced by McCulloch and Pitts in 1943^25^. Thank to the continuous development of such machine learning techniques, they can be applied successfully in ecology combined with other methods like classification, clustering, and recognition of patterns in non-linear species-environment relationships. Following the rapid development of computational techniques in the 1980s and 1990s, ANNs bacame widely applied in ecological studies as well. ANN is able to recognise community types^26–29^, phenological and developmental dynamics^30–34^, environmental changes^35,36^, and prediction of time series data^37–40^. In mycological studies, ANNs are commonly used for decision-making procedures such as theconsumptionability of different fungal species^41–43^, or for the identification and monitoring of ideal growing environment in growing halls^44,45^, nontheless for the molecular investigation of fungi as foods content and the quality determination^46–49^. However, as disscussed extensively in the critical review of Yin et al.^50^, the application of computer vision and ANN technologies in mushroom industry is contraversory. According to their work, most of the machine learning techniques have been developed to characterise and monitor certain identification and to define characteristics of fungi, such as colors^51^ and shapes^52,53^, stem cuts, how the universal veil opens^52^ and diameters of the pileus^55^. These attributes have already been successfully used in mushroom cultivation and harvesting^50^. Moreover, Verma et al.^42^ applied artificial neural networks and neuro-fuzzy inference systems to separate edible and inedible sporocarps.

Despite the fact that a number of studies have demonstrated that meteorological variables affect fungal development^42,50–54^, no attempt has been made so far to quantify or predict the occurrence of fungus in natural and undisturbed habitats using non-linear modeling. Therefore, we aimed to evaluate the possible fructification of some selected fungal species applying species-specific combinations of weather parameters using multilayer perceptrons (MLP) with backpropagation (BP) algorithm for supervised learning. *Russulaceae* and *Amanitaceae* families are important and widespread taxa in Hungary with edible, inedible and poisonous representatives as well. Hence, the prediction of their occurrence is of high importance in terms of both scientific and human safety reasons. As a result of this investigation, it became possible to estimate the occurrence of fruitbodies of certain *Russula* and *Amanita* species in temperate forests of western Hungary with moderate to high accuracy for the first time.

The formation of fruitbodies of each species is worth investigating individually because different temperature-water combinations induce their appearance according to their different ecological needs. There are few studies, especially for the economically important fungal species that examined the fruitbody formation of a particular species in a natural environment as a function of temperature and precipitation. For example, the water deficit in the soil due to dry summers, together with the high temperature, resulted in a decrease in fruitbody formation in some truffle (*Tuber*) species^55,56^. Our findings may open the possibility towards the integration of other fungal species into the estimation of their occurrence based on selected weather parameters with the highest R^2^ scores.

## Results

### ANN modeling of Amanita species

**Figure 1:**
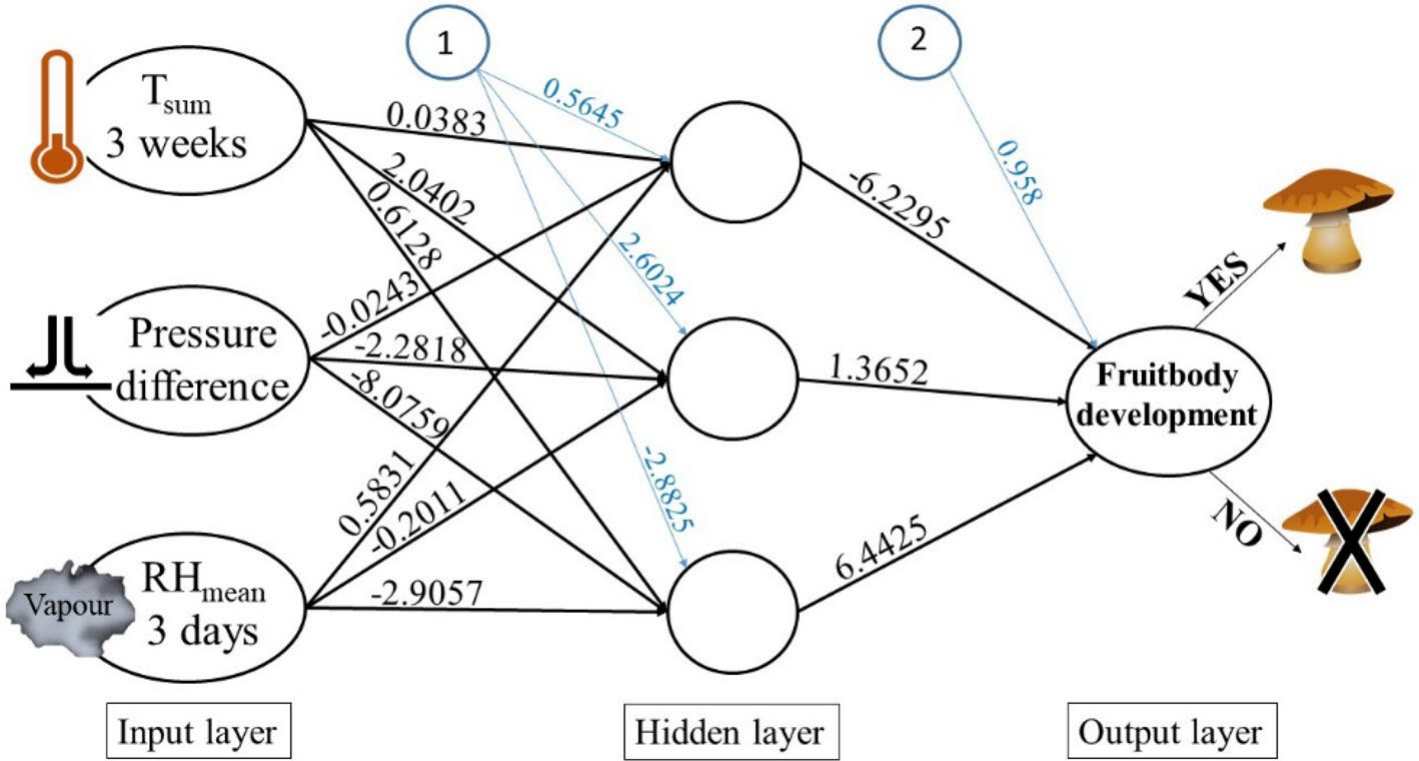
ANN model of *Amanita mairei* Foley with genus-level meteorological parameters (Tsum_3weeks, pressure difference and mean relative humidity for 3 days based on Table 2). Number of neurons in the hidden layer is 3. Error is 0.013274, steps in the hidden layer is 90. Blue lines represent the bias terms just like the intercept in a linear model.

The goal of this study is to apply a mathematical method (ANN) that is able to predict the output of a non-linear ecological system, in our case: fruitbody formation. Figure 1 shows the results of the ANN model of *A. mairei* with input weather parameters, nodes and calculated weights. The occurrence of *A. mairei* was calculated by applying genus-level meteorological variables, such as cumulative heat amount for 3 weeks, pressure difference, and mean relative humidity for 3 days (selection based on Table 2).

**Figure 2:**
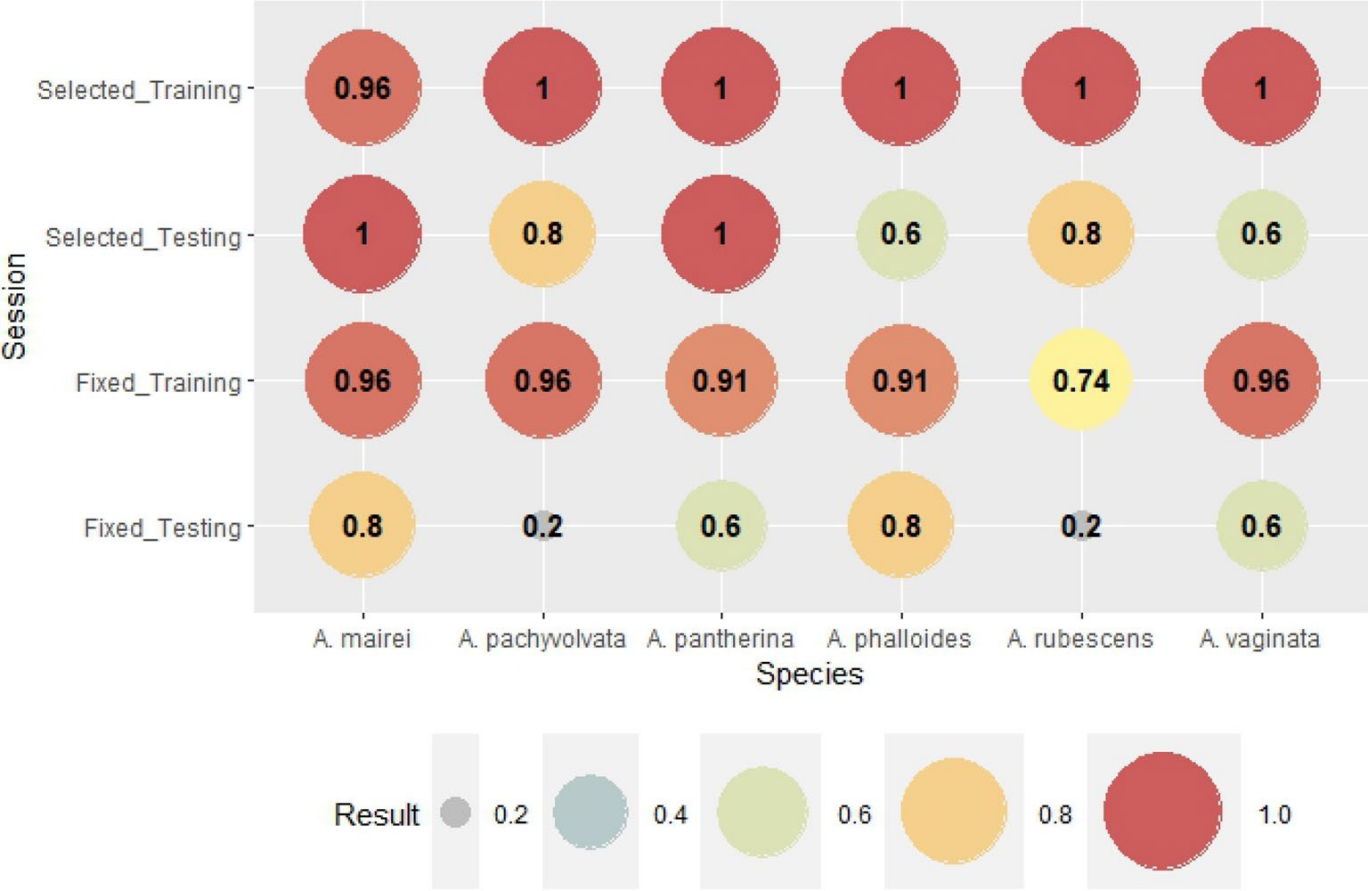
The rate of correct forecast (hit + correct negative, see Table 3) from all forecast data (hit + false alarm + missed + correct negative). The score is 1 for a perfect forecast.

**Figure 3:**
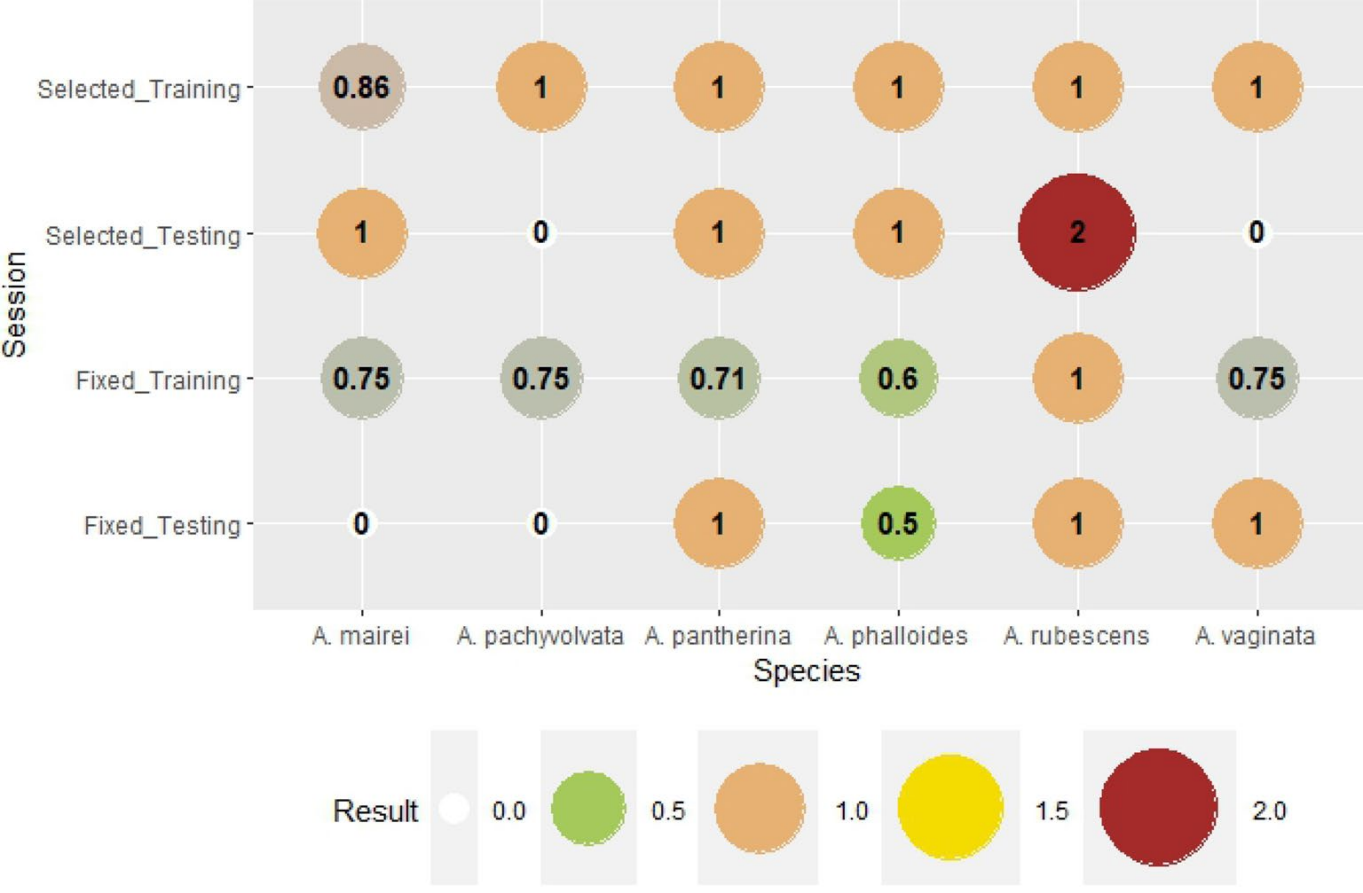
Bias is the rate of all forecasted (hit + false alarm) and all observed (hit + missed) fruitbody formation. If Bias is 1, the numbers of false alarm and missed forecast are equal, which can be seen as a perfect forecast if both number is zero. If Bias is greater than one, the fruitbody formation is overestimated. If bias is smaller than one, then the event is underestimated.

**Figure 4:**
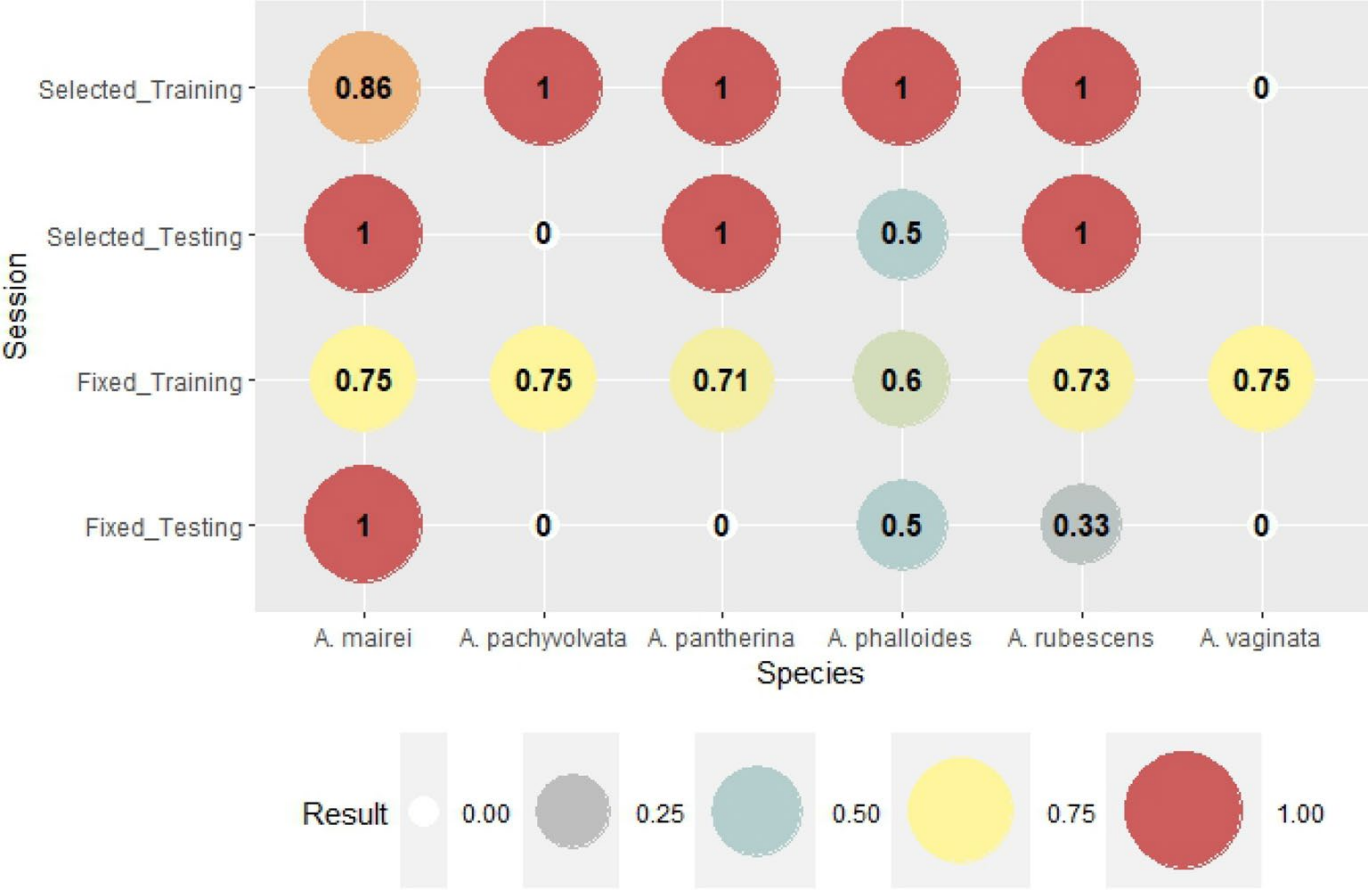
The rate of correct hit out of all fruitbody occurrence (hit + missed). If the probability is 1, i.e. the detection is 100%, then all of the fruitbody formation were correctly predicted. The cell is empty when the divisor was zero.

**Figure 5:**
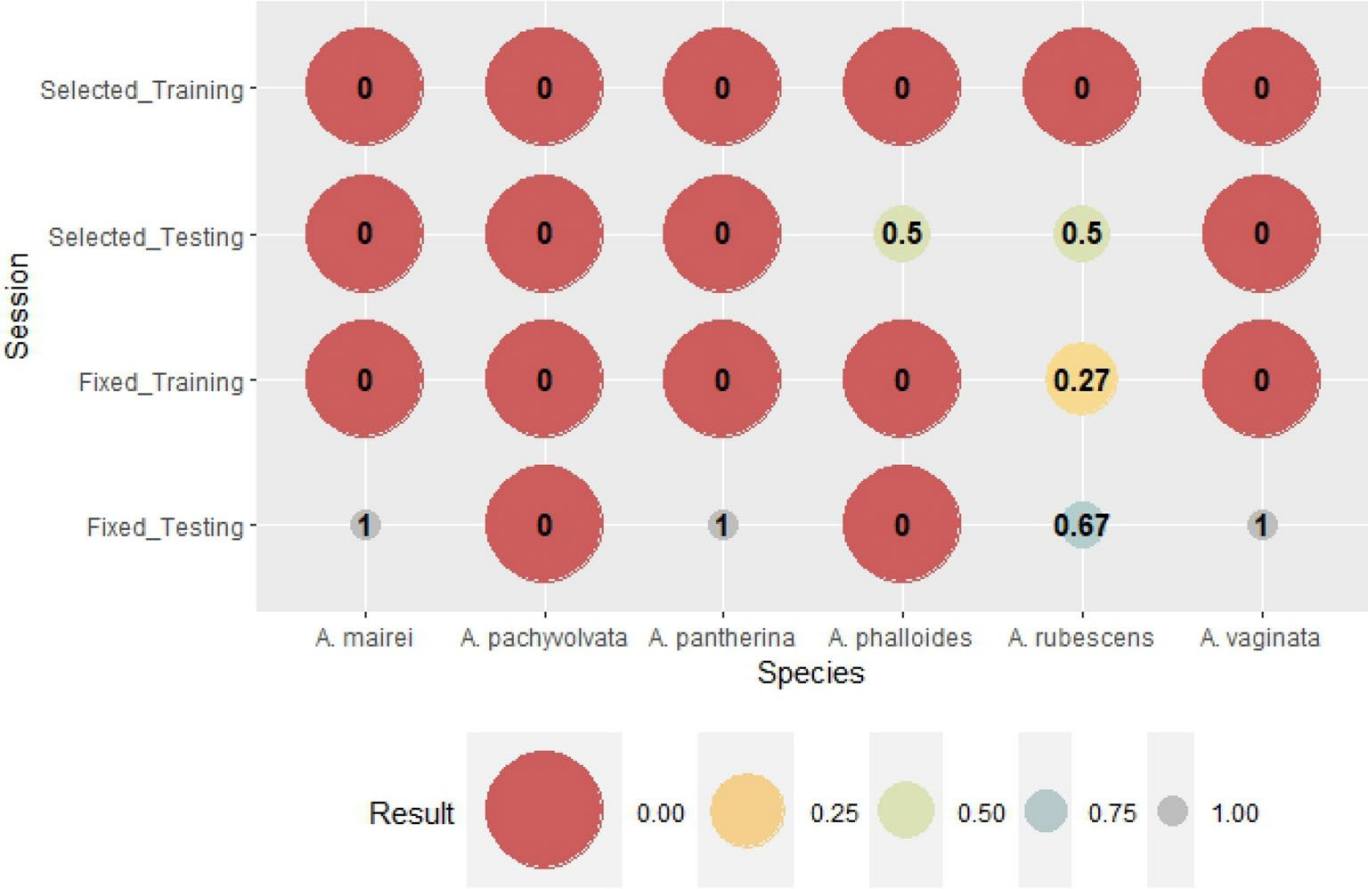
The rate of false detections out of the summarized value of hits and false alarms. If the index was zero, there was no false forecasts for fruitbody formation.

**Figure 6:**
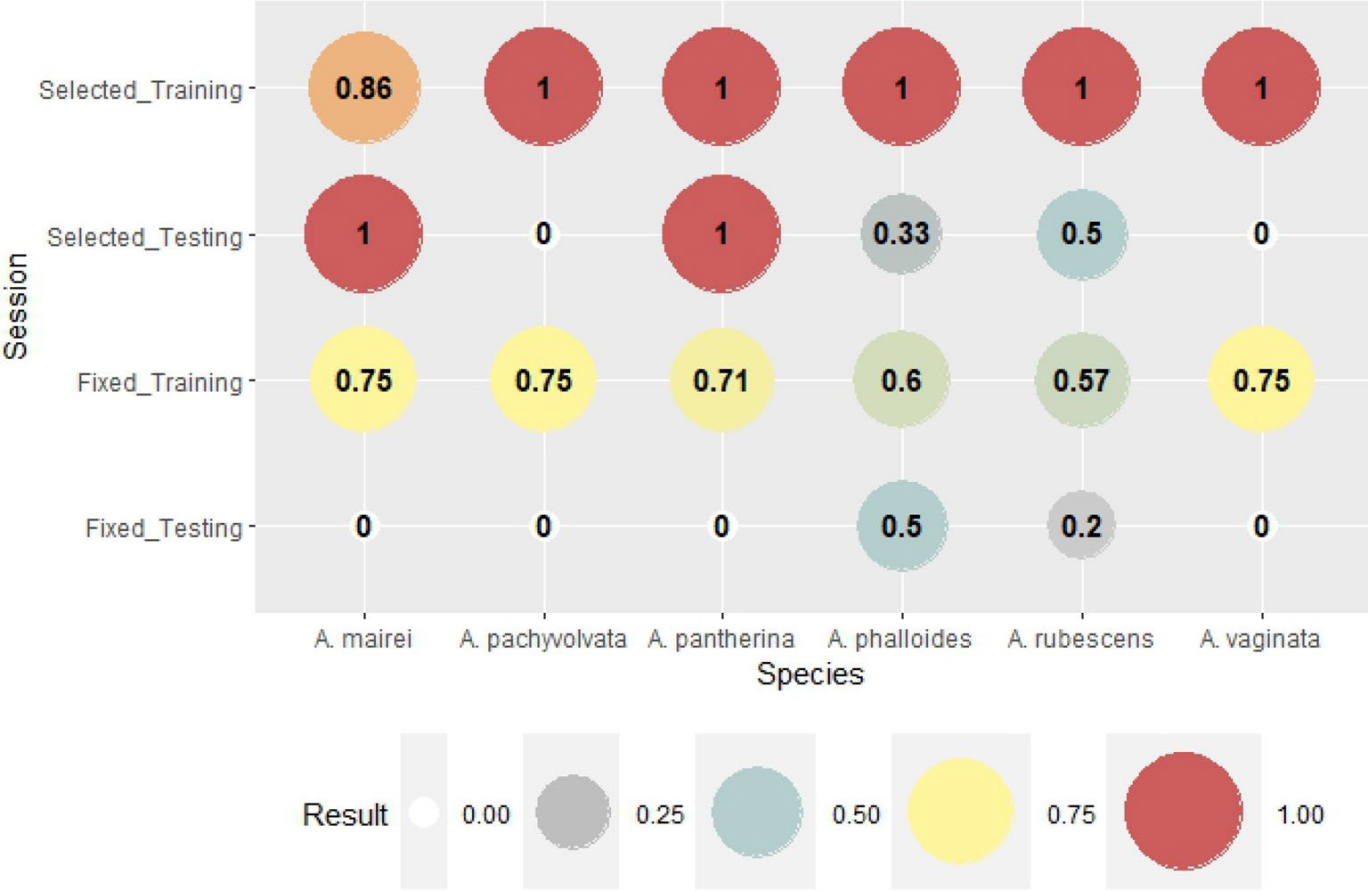
The rate of correct hits out of the summarized values of all correct, missed and false detections of fruitbody formation. In case of a perfect forecast, CSI is one.

**Figure 7:**
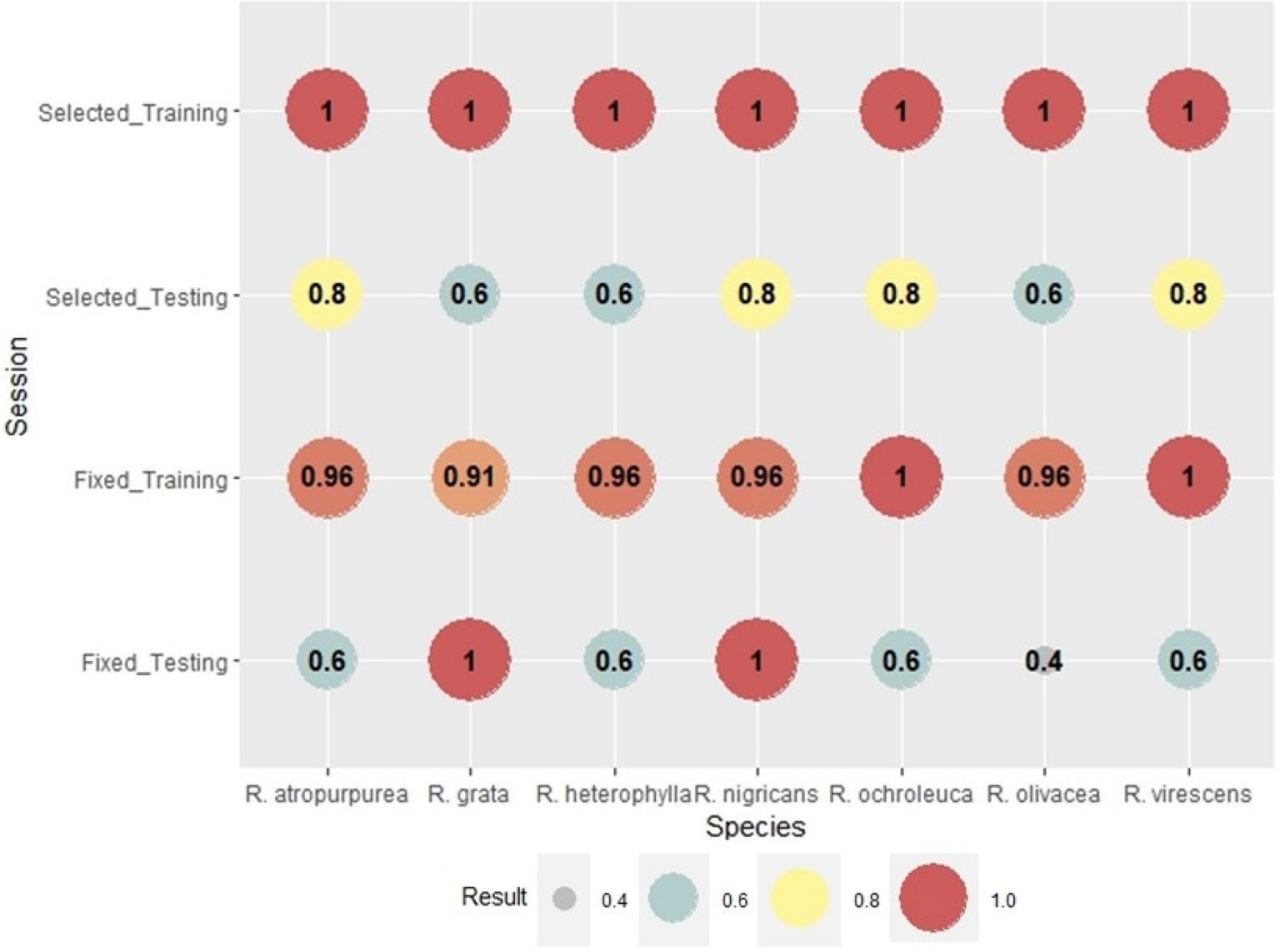
The rate of correct forecasts (hit + correct negative, see Table 3) out of all forecast data (hit + false alarm + missed + correct negative). The score is 1 for a perfect forecast.

**Figure 8:**
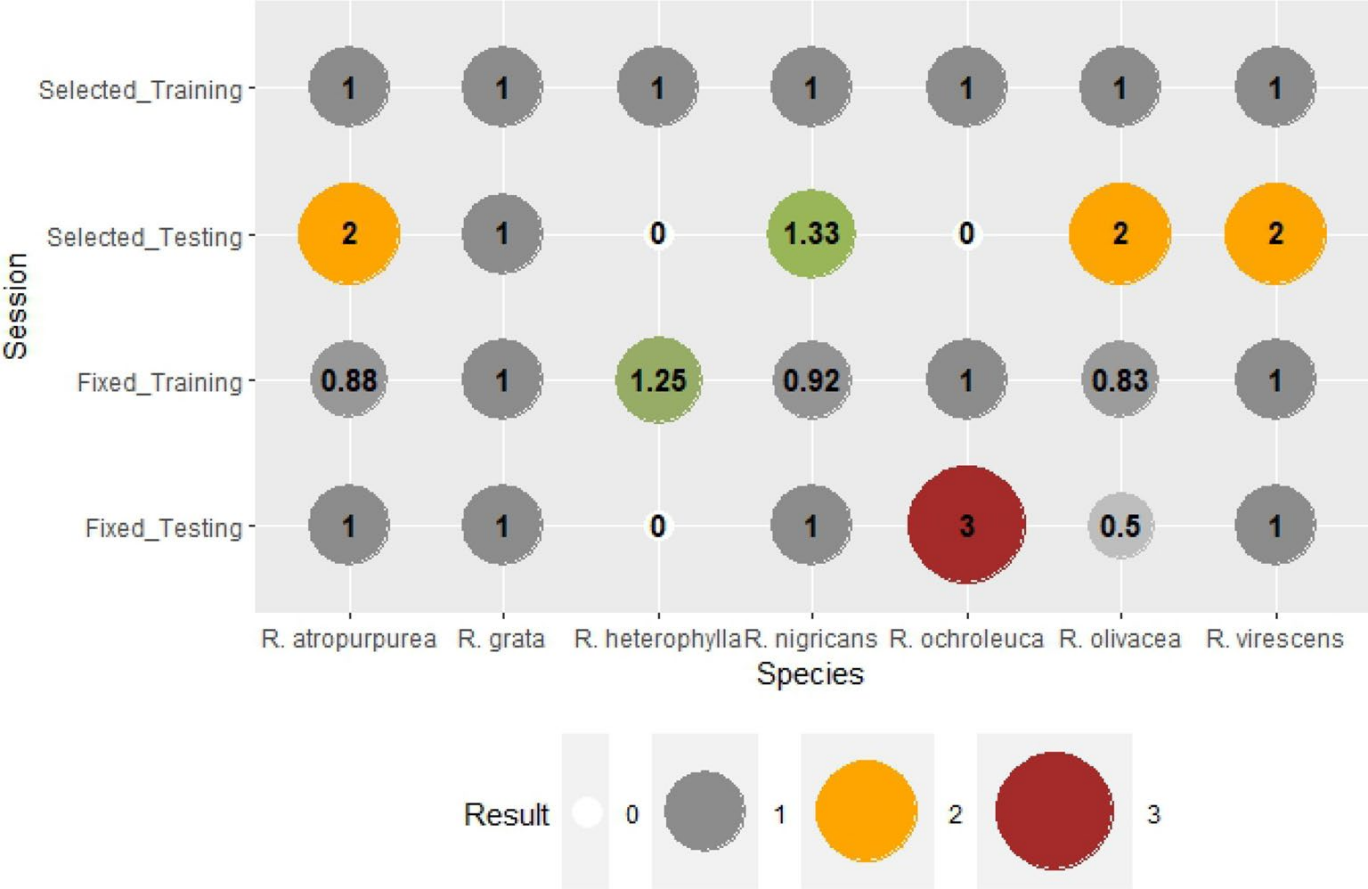
Bias is the rate of all forecasted (hit + false alarm) and all observed (hit + missed) fruitbody formation. If Bias is 1, the number of false alarms and missed forecasts is equal, which is seen as a perfect forecast if both numbers are zero. If Bias is greater than one, the fruitbody formation is overestimated. If bias is smaller than one, then the event is underestimated.

**Figure 9:**
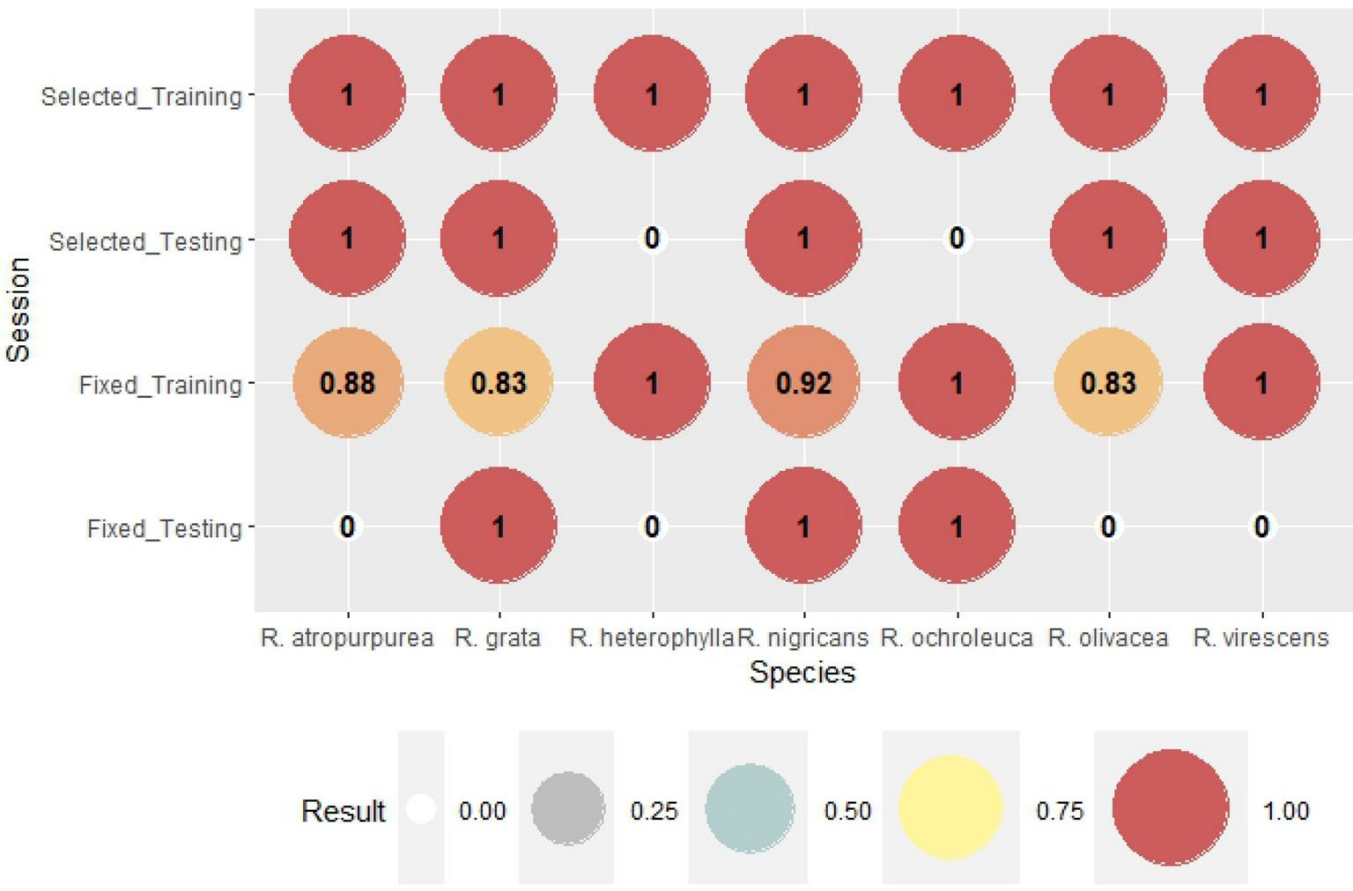
The rate of correct hits out of all fruitbody occurrences (hit + missed). If the probability was 1, i.e. the detection was 100%, which represented correctly predicted fruitbody formations.

**Figure 10:**
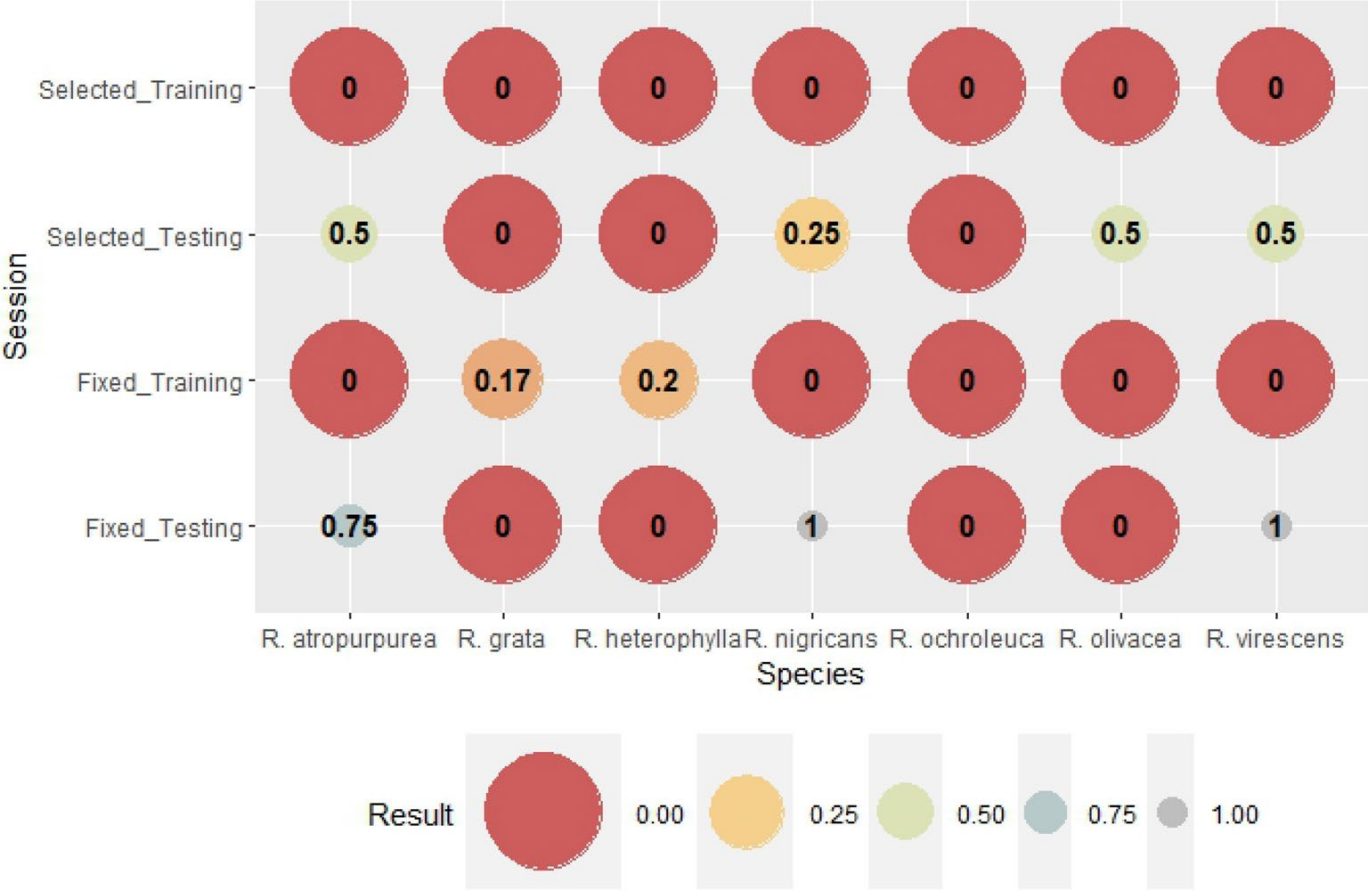
The rate of false detections out of the summarized value of hits and false alarms. If the index is zero, there was no false forecasts of fruitbody formation.

**Figure 11:**
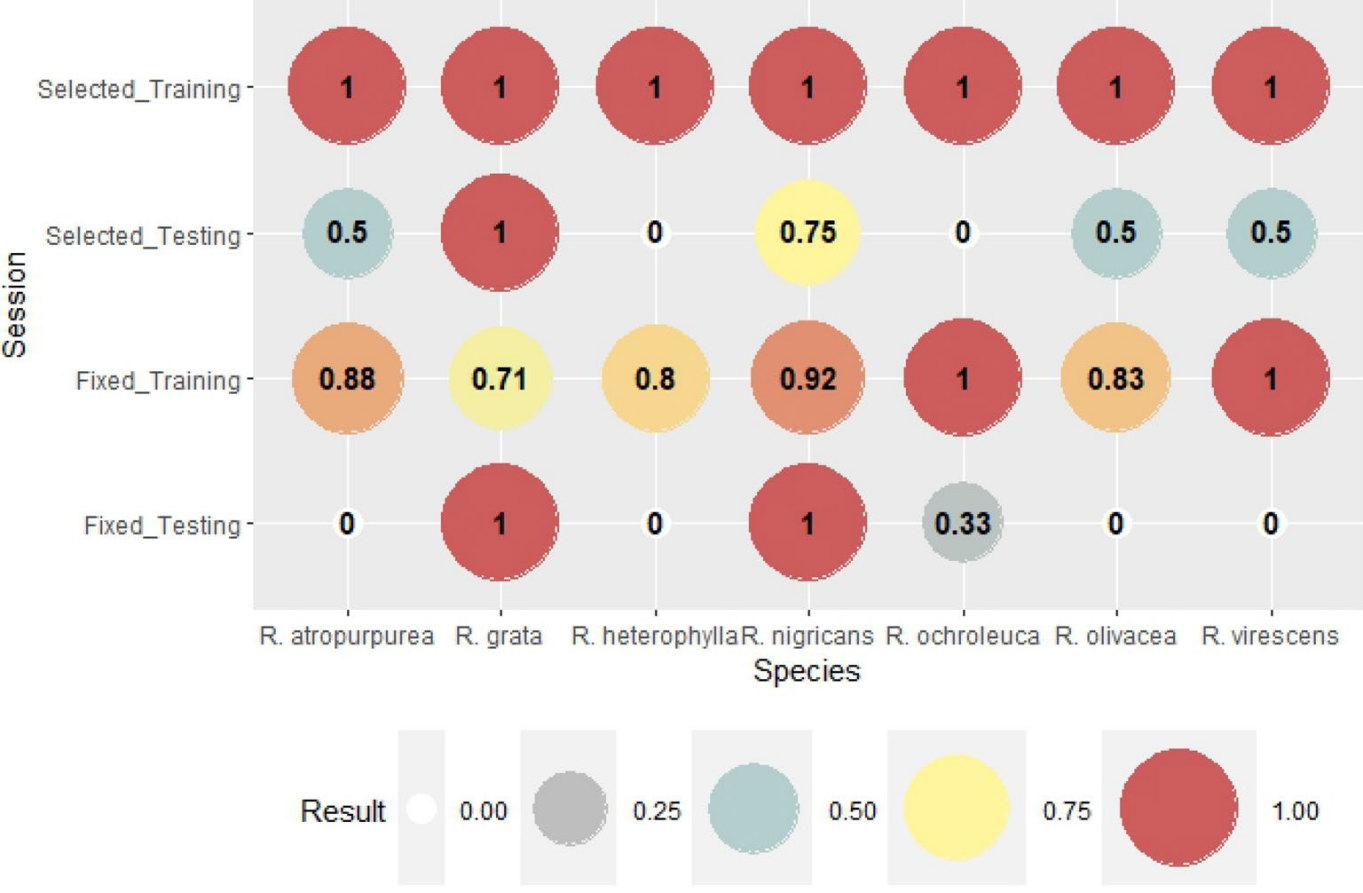
The rate of correct hits out of the summarized values of all correct, missed and false detections of fruitbody formations. For a perfect forecast, the CSI score is one.

The verification of the above mentioned ANN modeling for all species can be seen in Figs. 2, 3, 4, 5, 6, 7, 8, 9, 10, 11. Accuracy (Fig. 2) shows the rate of correct forecast (hit + correct negative) from all of the forecast data (hit + false alarm + missed + correct negative) (Table 3). The training session, where binomial outputs of fruitbody formation (Yes/No) were incorporated into the calculations, achieved high reliability of forecast, mostly above 0.9 in the case of genus-level meteorological variables (except for *A. rubescens*). Furthermore, there were mostly perfect forecasts (ACC = 1) for species-selected weather variables except for *A. mairei*, with a reliability of prediction of 96%. As it was expected, the testing session shows worse verification scores compared to that of the training session. However, a significant improvement can be observed in the case of forecasts based on species-selected weather parameters. The average accuracy score is 54% for predictions based on genus-level meteorological variables and 80% based on species-selected variables.

The next verification index is Bias (Fig. 3). Bias is the rate of all forecasted (hit + false alarm) and all observed (hit + missed) fruitbody formation. If Bias is 1, the number of false alarms and missed forecasts are equal, which can be seen as a perfect forecast if both numbers are zero. If Bias is greater than one, the fruitbody formation is overestimated. If the Bias is smaller than one, the event is underestimated. In the training session for species-specific weather variables, the Bias with scores of 1 reflects the perfect prediction of fruitbody formation, which is supported by the results in Fig. 3. (Accuracy is 100% for species-selected variables in the training session). There were two cases (genus-level variables in the testing session and species-specific variables in the testing session as well), when Bias was impossible to be interpreted mathematically because of zero divisors; it represents no fruitbody formation (neither hit, nor missed). Fruitbody formation was underestimated in cases of genus-level weather parameters and training sessions (except for *A. rubescens*, for which the number of missed forecasts and false alarms was equal). There was overestimation only in one case, in the species-selected testing session of *A. rubescens,* where the Bias was two.

The results of probability of detection (POD) index are presented in Fig. 4. POD is the rate of correct hits out of all fruitbody occurrences (hit + missed). If the probability is 1, i.e. the detection is 100%, all of the fruitbody formation was perfectly predicted. The difference between the results of fixed (genus-level) and selected weather parameters is obvious. While the detection rate in the case of genus-level variables for the training session was 70%–75% (60% for *A. phalloides*), the detection was 100% for most species in the training session (86% for *A. mairei*). The detection rate in the testing session for genus-level weather variables was very low even with 0% detection in three cases (*A. pachyvolvata, A. pantherina, A. vaginata*). The selection of weather parameters in the cases of species (no genus-fixed weather variables) significantly improved the detection rate (POD) with a 100% score for 3 out of 6 species (*A. mairei, A. pantherina, A. rubescens*). However, the detection of *A. pachyvolvata* was unsuccessful in both cases of the testing sessions.

False alarm ratio (FAR) is the rate of false detections out of the summarized value of hits and false alarms. If the index was zero, there were no false forecasts of fruitbody formation. The value of FAR was high in the cases of the testing session both for genus-level and species-specific weather parameters, especially for *A. rubescens* and *A. vaginata* (Fig. 5). There were no false forecasts in the training session of species-specific weather parameters, while a decrease of scores can be observed in the testing session. The average score of FAR for genus-level variables in the testing session was 0.611; for the selected variables in the testing session, it was 0.167; therefore, the false alarm ratio was significantly better in the case of species-specific weather parameters.

The critical success index (CSI) is the most rigorous index. It shows the rate of correct hits out of the summarized value of all correct, missed and false detections of fruitbody formation. For a perfect forecast, the score of CSI is one. The difference between the reliabilities of forecasts based on genus-level and species-specific weather parameters was the most obvious here (Fig. 6). The result of the training session for selected weather parameters reflects the perfect estimation of fruitbody formation (except *A. mairei* with 86%), while the CSI of the training session for genus-level variables varied among 0.57 and 0.75. The differences between the testing sessions were also significant. There were perfect forecasts for *A. mairei and A. pantherina; forecasts with CSI of 0.33 and 0.5 were characteristic of A. phalloides* and *A. rubescens*, respectively. There were no correct forecasts for *A. pachyvolvata* and *A. vaginata* (CSI = 0). In the case of genus-level weather parameters, there were only two species for which CSI was not zero (0.5 and 0.2 for *A. phalloides* and *A. rubescens*, respectively).

### ANN modeling of Russula species

The same verification indices were calculated for the results of ANN modeling of the fruitbody formation of *Russula* species. The accuracy of the forecast of *Russula* species is presented in Fig. 7. Calculation with genus-level meteorological variables showed an accuracy of 91% for *R. grata* and 96% for *R. atropurpurea, R. heterophylla, R. nigricans,* and *R. olivacea*. Perfect forecasts were performed for *R. ochroleuca* and *R. virescens*. In the cases of species-sepecific weather parameters, the Accuracy was 100% in all cases. The testing part of ANN modeling is always critical because this part of the process means the blind forecast of the fungal appearance. The lowest Accuracy score was 40% in the case of genus-level weather variables and for *R. olivacea*. The average score in this group was 68.57%. The lowest score was slightly higher (60%) in the cases of selected variables, testing part. The average score of Accuracy was 71.43%.

Bias indicates the over- or the underestimation of the model (Fig. 8). The fruitbody formation of *Russula* species in the case of genus-level variables, training session was underestimated for *R. atropurpurea* (0.88)*, R. nigricans* (0.92)*, R. olivacea* (0.83) and overestimated for *R. heterophylla* (1.25). There was no significant difference (BIAS = 1) for *R. grata*, *R. ochroleuca,* and *R. virescens*. The Bias of the training session for species-specific weather parameters was 1 for all *Russula* species. The testing session shows the blind forecasts, i.e. using all the weights calculated in the training session for the prediction of fruitbody formation. In the case of genus-level variables the Bias was more balanced. Bias was 1 for 4 out of 7 species (Fig. 8), there was underestimation in two cases (*R. heterophylla, R. olivacea*, B = 0.0 and B = 0.50, respectively). A significant overestimation for *R. ochroleuca* was also observed (B = 3.0). In the training session for species-selected weather variables, the Bias was increased and overestimation was seen for *R. antropurpurea, R. nigricans, R. olivacea,* and *R. virescens*. Underestimation was detected for *R. heterophylla* and *R. ochroleuca*. The only species where Bias was 1 in all training and testing groups was *R. grata*.

The scores of Probability of detection (POD) were higher for the species-selected variables than for the genus-level weather parameters (Fig. 9). In the training session, the lowest value, even for genus-level weather variables, was as high as 83%, which suggests high reliability of detection. In the training session for the species-specific weather variables, POD was 1 for all species, which means perfect detections of fruitbody formation. In the testing session, the detection takes only two values: 0 and 1. The detection of fruitbody formation in the case of genus-level weather variables was totally missed for *R. atropurpurea, R. heterophylla, R. olivacea,* and *R. virescens*. However, the detection was perfect for three species *(R. grata, R. nigricans*, and *R. ochroleuca*). The weather parameters for the species-specific cases showed a higher probability of detection; 5 out of 7 *Russula* species were detected perfectly (POD = 1). In the case of *R. heterophylla* none of the methods (genus-level or species-specific variables) were able to successfully detect the fruitbody formation.

False alarm ratio (FAR) was suitable to describe the false detections (Fig. 10). It was logically connected to the results of Bias (Fig. 8). When the fruitbody formation was overestimated, the number of false alarms should have been higher. In our study, nothing but false detections (FAR = 1) were found for *R. nigricans* and *R. virescens* for genus-level variables in the testing session. However the False alarm ratio was high for *R. atropurpurea* also (FAR = 0.75). If the index is zero, there were no false forecasts of fruitbody formation. *R. grata, R. heterophylla* and *R. ochroleuca* should be mentioned as there were no false detections in their testing sessions at all.

Critical success index (CSI) was taken into consideration for all the observed and forecasted occurrences of fruitbody formations. CSI was 1 when there were neither false alarms, nor missed hits. As it was seen for the other indices, the forecasts were perfect in cases of species-specific weather parameters during the training session (Fig. 11). CSI scores increased for 3 out of 7 species and decreased for 2 out of 7 species during the testing session, contrasting the methods of genus-level and species-specific weather parameters. CSI increased for *R. antropurpurea*, *R. olivacea*, and *R. virescens*, while decreased for *R. nigricans* and *R. ochroleuca*. No change in CSI scores was observed for *Russula grata* and *R. heterophylla*.

Based on our results, it can be concluded that the ANN properly works for forecasting the appearance of mycorrhizal groups. However, we do not know how it would work for xylophagous, soil saprotrophic species, since they live in a different ecological niche. Furthermore, in this study, we do not deal with small groups such as coprotrophs, species living on herbaceous plant remains, parasites on insect pupae etc., because there is not enough data available for a sufficient statistical analysis.

The study and the presented results have practical importance, since the ANN method is able to forecast the possible occurrence of the fruitbody using actual weather data. These meteorological parameters are widely available and easy to calculate, so they need low costs and computational demand. In comparison to camera-based systems, such as CCD-type cameras, which have very high acquisition costs due to their exceptional sensitivity. Moreover, camera-based analysis is limited in scope, as images can only be captured in locations where cameras are deployed. In the future, the above-presented ANN-based fruitbody estimation technology may play a vital role in overcoming former limitations.

Nevertheless, a more frequent, i.e. weekly forecast of fruitbody formation may significantly speed up the ongoing mycological researches, as it increases the chances of collecting a given taxon faster.

Since fruitbody formation data stems from two experimental sites with very similar macroclimatic background, therefore in order to broaden the applicability of ANN, it is recommended to include more locations. Also, in the future we will extend the investigations to the fruitbody formation of species belonging to other functional groups (soil saprotrophic, xylophagous etc.) in order to find out for which groups the method can still be applied. Lastly, as a further extension of the scope of ANN applicability, it can be inferred from the above-presented results that it is suitable for clarifying taxonomic problems, i.e. for sorting out species and species complexes that have not yet been fully known and characterized.

## Discussion

Based on the results of this study, the current work is novel in its field from different perspectives. First, to the best of our knowledge, this study is the first to introduce their application for evaluating the formation of macrofungal fruiting bodies in natural and undisturbed habitats. Moreover, only a handful of fungal species, including *Agaricus bisposrus*^57^, *Oudemansiella raphanipes*, and *Pleurotus eryngii*^58^, have been studied previously; however, the analyses presented in this work represent the first ANN application results for *Russula* and *Amanita* species.

Learning algorithms have been mostly applied in mycology for decision-making processes such as the recognition of fungi by images^43^, the consumability of fungi^59,60^, or control systems for mushroom farms^61^. In the work of Hernández-Rodríguez et al.^62^, vegetation characteristics and their effect on the occurrence prediction of king mushroom (*Boletus edulis*) were investigated. They found that the mean height of the scrub vegetation was the most reliable parameter for the prediction of fruitbody production^62^. Furthermore, Solanki et al.^63^ conducted a survey on weather prerequisites for the fructification of *Phellorinia* species, but this work only utilized air temperature, relative humidity and total rainfall. From these data, they attempted to find correlation among the weather parameters and fruitbody occurrence (26^th^ to 36^th^ calendar weeks) and from these results, they identified the most probable starting date of the initiation and termination of fruitbody formation (23^th^ to 39^th^ calendar weeks) as the most suitable conditions to conduct domestication experiments^63^. Although the role of weather and vegetation conditions on fruitbody formation is extensively studied^62–64^, ANN models have not yet been applied and verified for the prediction of fruitbody formation.

There is a significant effect of air pressure and temperature on the fruitbody formation for *Amanita* species, and it is generally characteristic for all studied species of *Amanita* genera. In order to extend this conclusion, it would be beneficial to increase the number of studied *Amanita* species in the future.

The prediction of fruitbody formation based on genus-level, meteorological variables for *Amanita* species was less reliable than species-specific weather parameters. The ANN model achieved two perfect forecasts, namely for *A. mairei* and *A. pantherina*. Another noteworthy result was achieved in the case of *A. rubescens*, which can be estimated with 80% accuracy and 100% probability of detection, however there is an overestimation in the model. The less reliable estimation occurred for *A. pachyvolvata* and *A. vaginata,* because of the lack of successful fruitbody forecast, i.e. the model did not predict any occurrence of fruitbody formation in any case. An explanation for these diverse results may be that the species complex is large, the taxonomic classification of the species is unclear^65,66^, and therefore, it is still in progress^67–69^.

The influence of environmental factors was much more diverse for *Russula* species than for *Amanita*s, which was also reflected in the selection of genus-level weather parameters and their correlations to fruiting body formation.

As for the testing session of the *Russula* genera, the results of the verification indices using genus-level weather variables were of a higher standard than anticipated from the *Amanita* species. In the case of two species, namely *R. grata* and *R. nigricans*, the forecast of fruitbody formation was perfect. In addition, *R. ochroleuca* may be mentioned with moderate scores of accuracy and with significant overestimation of occurrences. Other species from *Russula* genera had very low scores and weak forecasting reliability. In the case of species-specific weather parameters, the results showed that verification indices had lower standard deviations compared to the genus-level meteorological variables. The reliability of the estimation of fruitbody formation was the highest for *R. grata*, high for 3 out of the 7 *Russula* species (*R. nigricans*, *R. antropurpurea,* and *R. virescens*), and moderate for *R. olivacea*. The estimation of *R. heterophylla* and *R. ochroleuca* was the least reliable. All in all, the number of successful predictions was 3 out of 7 species in the case of using genus-level weather parameters and 5 out of 7 species for species-specific meteorology. In those cases, where neither genus-level, nor species-selected variables were successful in the prediction of fruitbody formation (e.g. *R. heterophylla* and *A. pachyvolvata*), other possible internal or external effects must presumably exist which can have more significant influence on fungal formation than meteorology.

It may be inferred from the outcome of the investigation that artificial neural network (ANN) is able to predict the occurrence of mycorrhizal groups with medium to high probability. The functioning of xylophagous, soil saprotrophic organisms in a distinct ecological niche remains unknown. Due to insufficient data for statistical analysis, we do not do research on small groups such as coprotrophs, species that rely on herbaceous plant remnants for survival, or parasites that inhabit insect pupae.

The findings indicate that the approach employed in this study may not be universally applicable for forecasting the emergence of fruiting bodies, especially in the case of mycorrhizal fungi species with a notably brief growth period. Examples of such species include the winter truffle (*Tuber brumale*), which has a brief fruiting phase lasting only two months during the winter and mycorrhizal coralline fungi, such as the greening coral (*Ramaria abietina*), that exclusively generates reproductive structures during the fall season. Also, the applicability of ANN on xylophagous, soil saprotrophic organisms remains uncertain due to their occupation of a distinct ecological niche. In addition, this study does not include analysis of small groups such as coprotrophs, species that live on herbaceous plant remnants, or parasites on insect pupae. This is due to insufficient data for a comprehensive statistical analysis.

The limitations of the results of ANN can originate from the lack of precise in-situ microclimatic measurements of meteorological variables, although, due to the random nature of the appearance of the fungal bodies, it is not possible to satisfy this condition even with today's technological solutions. Eliminating this deficiency is one of the main reasons for using ANNs. The reliability of forecasting fruitbody formation can be improved by more detailed environmental data, such as soil parameters, ecological interactions among Amanita and Russula species and other present species as well. However, the goal of this study was to use easily accessible meteorological data to forecast fruitbody formation and verify the reliability of these predictions.

Another source of possible error connected to ANN has to be mentioned: the so-called overfitting of the model. This means that there are more weights than data. This problem has been alluded to in the manuscript, and care has been taken to ensure that the number of input data and the number of nods are proportional.

Our results demonstrate that artificial neural networks can be successfully applied to forecast fruitbody formation of selected *Amanita* and *Russula* species. Given our findings, it is recommended to analyze each species separately rather than by genera when studying the growth of fruitbodies induced by favourable meteorological circumstances. This is first demonstrated by the presentation of correlation coefficients among weather parameters and fruitbody occurrence data, where the relationship was significantly stronger between species-specific weather parameters and fruitbody occurrence than in the cases of genus-level weather variables. Moreover, the application of species-selected weather parameters enhanced the reliability of the ANN forecast of fruitbody appearance in most cases. The testing session can be considered a blind forecast, so the importance of these results is high and requires a more detailed examination.

Nevertheless, our findings indicate that this approach can effectively be employed for species with extended periods of growth, as those used in this study. Also in the future, it is worthwhile to investigate and evaluate the prediction of fruitbodí occurrence of webcap (*Cortinarius* ssp.), milk-cap (*Lactarius* spp.), and fibercap (*Inocybe* spp.) species.

## Materials and methods

**Figure 12:**
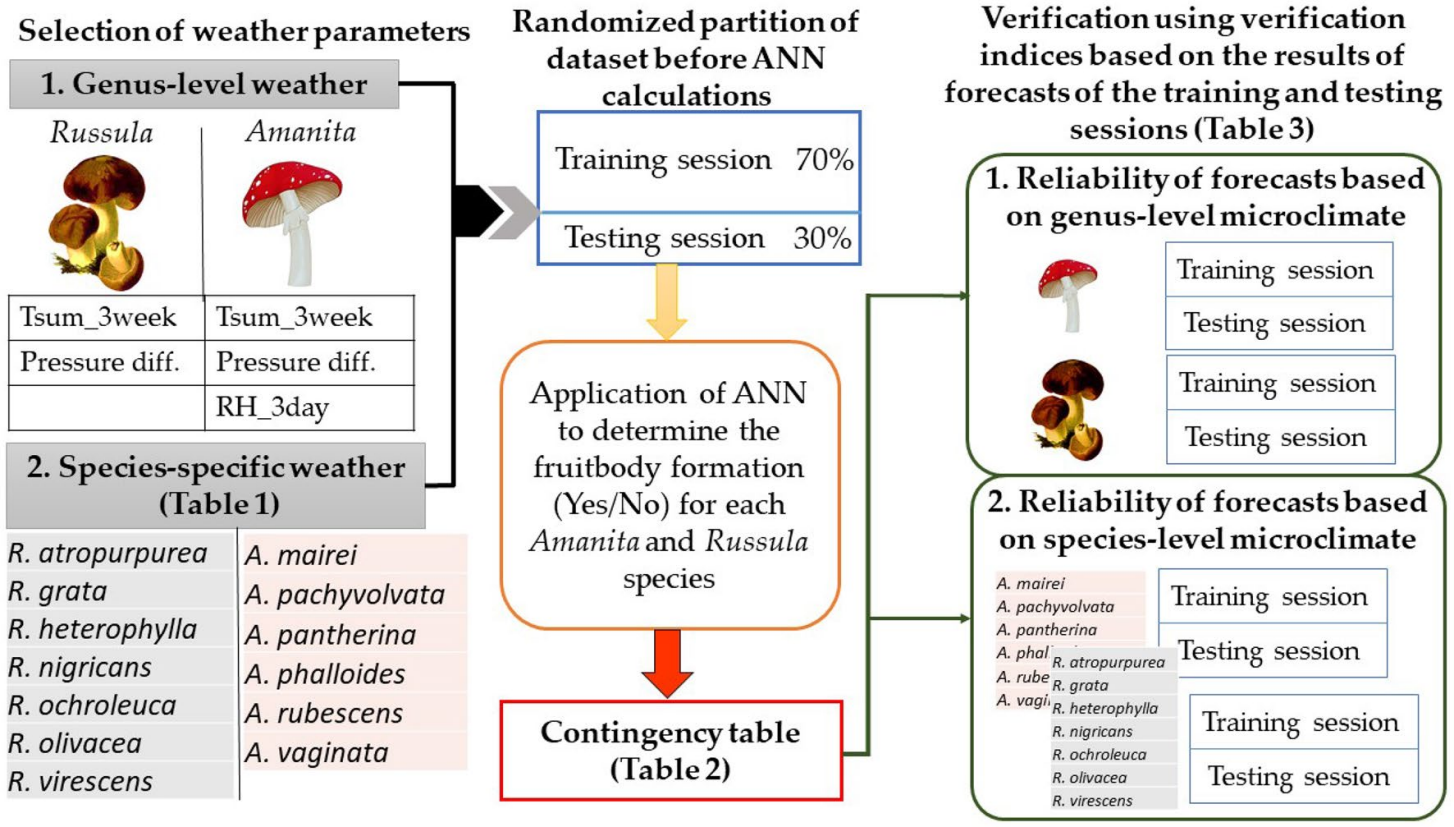
The flow chart of our work ranging from the weather parameters’ selection to the presentation of the results of the verification indices.

The structure of our work is presented in Fig. 12. Firstly, those weather parameters were determined on genus-level and species-specific levels, which showed the highest correlations with fruitbody formation. As a second step, the dataset in both cases was partitioned randomly into two parts containing 70% and 30% of the data, which are called the training session and the testing session, respectively. The artificial neural network, as a learning algorithm, used the training session to determine the best approximation of macrofungal formation as a binomial output (Yes/No) based on the weather parameters. The testing session was used to obtain real forecasts as the results of the non-linear algorithm acquired by the ANN. In the last but considerable step, the verification of the results of the training and testing sessions was achieved to prove the reliability of the forecasts.

### Experimental areas

Vendvidék is the westernmost area of Hungary and also of the Carpathian Basin. The region is a "mushroom paradise," where most macrofungi species fructify in the country due to its unique climatic and geographical features as well as its nearly complete forest cover. Consequently, the mycological literature is extensive, and their study in the field is thorough. Siller et al.^70^ published the distribution of protected macrofungal taxa, while Siller et al.^71^ reported an annotated checklist of hundreds of macrofungal species in the region. Connectedly, a current ecological study^72^ was also released. The sample size is around 2 km^2^. The area is mosaic-like, with roads and settlements, and consists of 3 forest blocks. The forest stands here are homogeneous with mixed forests of oak, beech, hornbeam, birch, Scots pine, spruce and alder.

**Figure 13:**
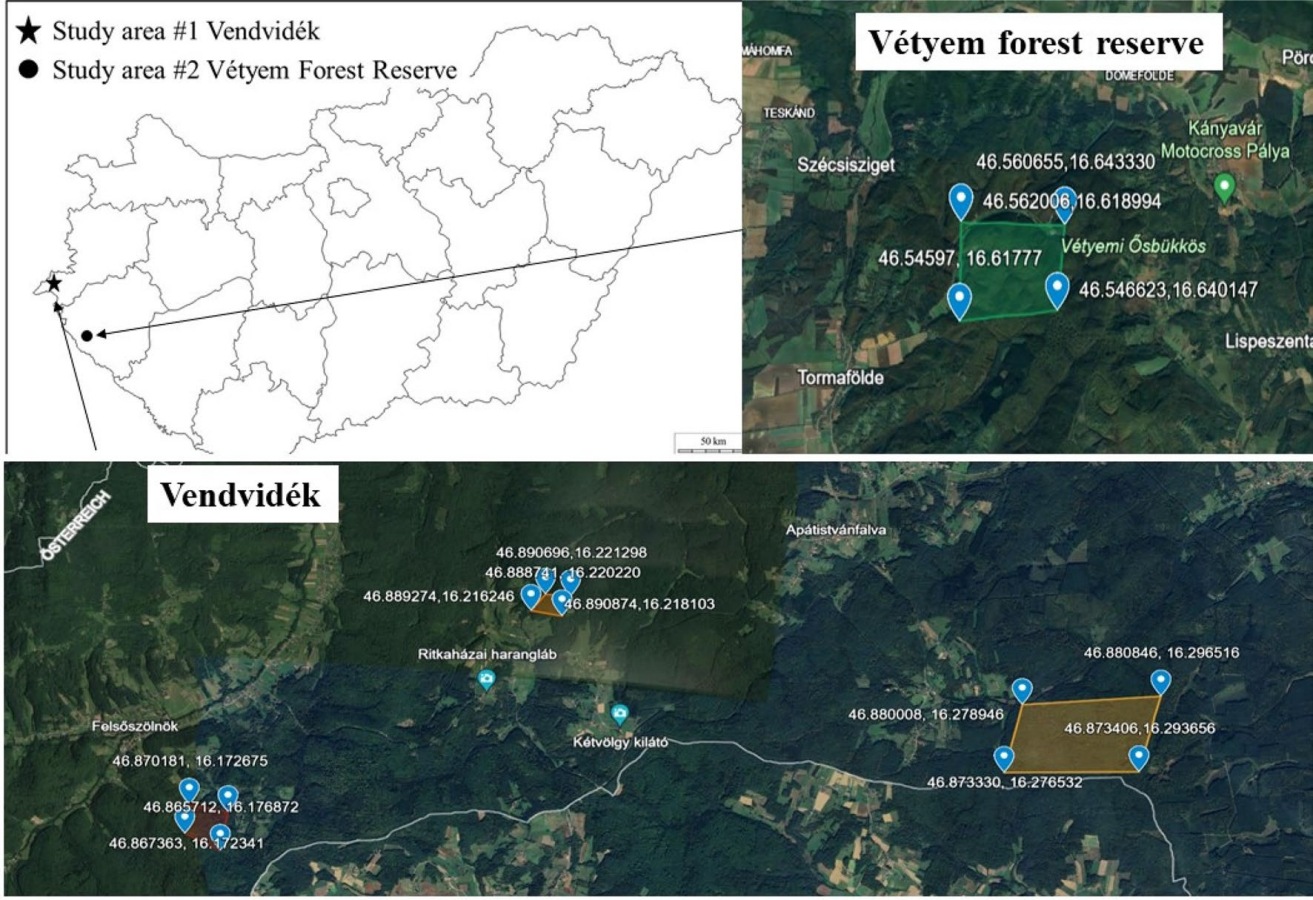
Locations of the two study areas in Vendvidék and in Vétyem forest reserve, western Hungary.

The Vétyem forest reserve is one of the oldest forest stands in Hungary lacking any forest management activities. Detailed mycological research of the area began in 2006 in the framework of the Hungarian Biodiversity Monitoring System^73^ and based on its protocol in the core area, buffer zone and one plantation of the forest reserve. This Forest Reserve is a forest block of 2 km^2^. It is mainly composed of hornbeam-oak and hornbeam-beech forest stands, but all important mycorrhizal partners are present, like birch, Scots pine, spruce, chert oak, and sessile oak. Vendvidék and Vétyem forest reserve are shown in Fig. 13 with the centre coordinates of N46.89°E16.23° and N46.5°E16.6°, respectively.

### Studied species

As long-term monitoring of fungi is ongoing in both areas, the number of documented macrofungal species in the two areas so far exceeds 600 species, which are being processed and published in the close future. For the species selected for this study, several important criteria had to be taken into account. Most importantly, they had to be exclusively associated with forest habitat, as the surveys were carried out in the forest. It was therefore obvious that mycorrhizal species had to be selected. In addition, the presence of a wide range of mycorrhizal partners in both areas (both deciduous and coniferous tree species) was an important factor. Another important factor was the longest possible growing season, i.e. the potential for the species to develop a growing season from summer to late autumn. The next criterion was to have occurrence data from both sites. Finally, it was important to have sufficient occurrence data for analysis. Because of these criteria, most documented species were eliminated, with selected species of the genera *Amanita* and *Russula* proving to be the most ideal. These species are also known to be important edible and toxic species in Hungary.

So the four criteria of the selection were:

- the species should be associated with forest habitats;
- the species must have a long fruiting period;
- they must occur at both locations at least ones due to the randomized partitioning of data for statistical analysis;
- they had sufficiently enough occurrence data for the analysis, so fruitbody formation must be occurred at least on 15% out of the 28 survey days

Thus, 6 species from the genus *Amanita* and 7 species from the genus *Russula* were included in this study, namely *Amanita mairei* Foley; *Amanita pachyvolvata* (Bon) Krieglst.; *Amanita pantherina* (DC.) Krombh.; *Amanita phalloides* (Vaill. ex Fr.) Link; *Amanita rubescens* Pers.; *Amanita vaginata* (Bull.) Lam.; *Russula antropurpurea* (Krombh.) Britzelm.; *Russula grata* Britzelm.; *Russula heterophylla* (Fr.) Fr.; *Russula nigricans* Fr.; *Russula ochroleuca* Fr.; *Russula olivacea* (Schaeff.) Fr.; and *Russula virescens* (Schaeff.) Fr.

### Field surveys

Twenty-eight survey days were chosen, 15 days of which in Vendvidék between 2018 and 2020 and 13 days in Vétyem forest reserve between 2016 and 2020 (Table 1).

**Table 1:** List of survey days in Vendvidék and in Vétyem forest reserve.

Vendvidék	Vétyem FR
12. 06. 2018	02. 09. 2016
25. 06. 2018	23. 06. 2017
20. 09. 2018	03. 09. 2017
01. 11. 2018	03. 11. 2017
15. 11. 2018	02. 09. 2018
17. 06. 2019	26. 10. 2018
29. 06. 2019	09. 11. 2018
19. 09. 2019	08. 09. 2019
29. 09. 2019	28. 09. 2019
10. 10. 2019	10. 11. 2019
12. 06. 2020	26. 09. 2020
16. 07. 2020	30. 10. 2020
27. 09. 2020	08. 11. 2020
22. 10. 2020	
07. 11. 2020	

Sampling was carried out during the main growing season from mid-summer to late autumn. At each sampling event, a complete field survey was carried out to find all species that were currently producing fruiting bodies. Species were identified from genus monographs and nomenclature was based on the Index Fungorum (January 2023). The number of fruitbodies was not directly recorded, but in all cases, the number of registered fruitbodies exceeded the one. On the other hand, the appearance of a large (> 100/ha) number of fruitbodies of particular mycorrhizal species usually occurs when some kind of extreme environmental impact affects the given species (e.g. deforestation, road construction, breaking of the waterproof layer of the soil). It can be stated that extreme human interventions did not occur in any of the areas in the examined time period, so there was no mass fruitbody production as a result of stress.

The lifespan of the fruitbodies of the examined species is 5–6 days on average. Of this, 3 days is considered a 'fresh' state, when most of the spore formation and spore emission takes place^74^.

**Figure 14:**
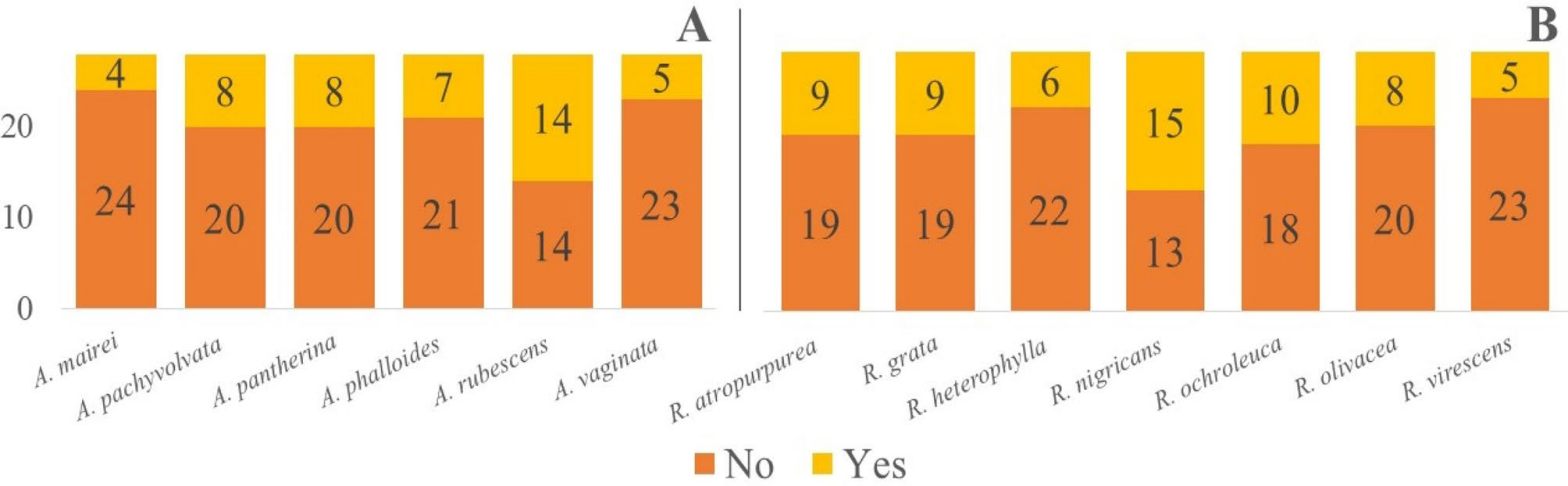
Number of days of sporocarp occurrences (Yes or No) out of the 28 survey days for (**A**) *Amanita* species; (**B**) *Russula* species. The output of occurrences is binary (1 or 0), e.g. in the case of *Amanita* mairei there were found fruitbodies 4 times out of the 28 survey days and there were not found any fruitodies 24 times out of the 28 survey days. The exact number of fruitbodies were not used for the calculations. The sample size (28 fungal data per ANN calculation) was appropriate for the ANN method (Haykin, 2009).

The fungus observations from the two locations were merged owing to the similar climatic conditions, therefore the sample size for the ANN models was overall 364 (13 species × 28 survey days) for *Russula* and *Amanita* species. The detailed number of occurrences can be seen in Fig. 14. The sample size for each species (i.e. the 28 data per species) was appropriate for ANN calculations^75^.

### Weather data

The weather data was obtained from the homogenized (i.e. removing the effects of the non-climatic factors, which can affect the measurement procedure, e.g. changes of instruments, data recovery procedures, etc.) climate data series of the Hungarian Meteorological Service. The dataset was interpolated to grid points using MISH v1.03 software^76^. The temporal resolution was 24 h, and the spatial resolution was 0.1° × 0.1°. In Vendvidék the nearest grid point was located in the middle of the study area with the coordinates of N46.5°E16.6°. In the Vétyem forest reserve, the coordinates of the nearest grid point were N46.9°E16.2°, approximately 5 km away from the study area (N46.89°E16.23°). Both study areas had subalpine climates with an annual mean temperature of 9–10 °C, annual precipitation of 800–850 mm, with a relatively high annual mean relative air humidity of 78%. The Köppen-Geiger climate classification is Dfb (humid continental climate)^77^.

Based on the climate series data mentioned above, 19 meteorological parameters were calculated based on daily mean temperature (°C), daily mean relative air humidity (%), daily mean air pressure (hPa) and daily precipitation (mm). The derived input parameters are mean temperature for 1 week, 2 weeks, 3 weeks, 4 weeks, cumulative heat amount for 1 week, 2 weeks, 3 weeks, 4 weeks, sum of precipitation for 1 week, 2 weeks, 3 weeks, 4 weeks ($\sum_{number\; of\; days}Pdaily)$, mean relative air humidity for 1 day, 3 days, 1 week, 2 weeks, 3 weeks, 4 weeks, and air pressure difference between two days in a row. The reference day was the survey day for all calculated microclimatic parameters in all cases. Means for a certain time interval were calculated by averaging the daily mean values of temperature or relative humidity for the given time interval, i.e. $\frac{\sum Tdaily}{number\; of\; days}$ for mean temperature and $\frac{\sum RHdaily}{number\; of\; days}$ for relative humidity. Accumulated heat amount is calculated by summarizing the daily mean temperatures for the given time intervals, i.e. $\sum_{number\; of\; days}Tdaily$. Owing to the climate characteristics of the study areas, negative values of daily mean temperatures were not observed on any of the survey days.

The selection of weather parameters to calculate ANN was performed by applying Pearson's correlations^78^ for each pair of weather parameters and mushroom species in order to select the most significant weather parameters driving fruitbody formation.

The main principles for selecting weather parameters were the following:

1. If the R^2^ > 0.5 for at least one weather variable, then the two weather parameters with the highest R^2^ scores were selected, each is one of a kind in order to avoid duplication of weather parameters with the same origin (i.e. temperature-based parameters, precipitation-based parameters, humidity-based parameters). The mean temperature and accumulated heat amount were considered to be of different origin based on the different calculation methods.
2. If the R^2^ ≤ 0.5 for all weather variables, then the three weather parameters with the highest R^2^ scores were selected, each is one of a kind (see above).

Increasing the number of input weather parameters did not increase the efficiency of ANN calculations as well as the stability and reliability of the forecasts, but instead, it led to increased computational demands. Table 2 contains the genus-level and species-specific meteorological variables for *Amanita* and *Russula* species. In the case of genus-level meteorological parameters for *Russula* species, the R^2^ scores were so low (R^2^ ≤ 0.1) that only two parameters were selected for ANN calculations.

**Table 2:** List of selected variables (cells with bold) and R^2^ scores for ANN calculation in the cases of genus-level weather parameters for all Amanita and Russula species and species-specific weather parameters selected by using the main principles decribed above. The whole list can be seen in Supplementary material (Tables 7, 8).

R^2^	Genus-level meteorology	Species-specific meteorology						
	All *Amanita* species	*A. mairei Foley*	*A. pachyvolvata (Bon) Krieglst*	*A. pantherina (DC.) Krombh*	*A. phalloides (Vaill. ex Fr.) Link*	*A. rubescens Pers*	*A. vaginata (Bull.) Lam*	
Tdaily_4week	0.032	0.045	**0.21**	− 0.235	0.064	0.129	**0.319**	
Tsum_3week	**0.224**	0.277	**0.143**	− **0.355**	**0.59**	**0.654**	0.075	
Pressure_difference	**0.199**	**0.607**	0.093	− **0.29**	**0.294**	**0.359**	**0.293**	
Psum_1week	0.089	**0.319**	0.158	− 0.018	0.191	0.144	− 0.065	
Psum_4week	0.097	0.077	**0.424**	− 0.128	0.028	0.091	0.027	
RH_3day	**0.117**	0.249	0.134	0.06	0.226	0.201	− 0.21	
RH_1week	− 0.013	0.041	− 0.086	0.187	0.033	− 0.073	− **0.357**	
RH_3week	0.039	− 0.012	− 0.08	**0.355**	0.016	0.01	− 0.253	

**R**^**2**^	All *Russula* species	*R. antropurpurea (Krombh.) Britzelm*	*R. grata Britzelm*	*R. heterophylla (Fr.) Fr*	*R. nigricans Fr*	*R. ochroleuca Fr*	*R. olivacea (Schaeff.) Fr*	*R. virescens (Schaeff.) Fr*
Tdaily_4week	0.073	− 0.002	0.257	**0.464**	0.191	− 0.592	− 0.051	**0.343**
Tsum_3week	**0.351**	**0.576**	**0.708**	0.415	**0.594**	0.397	**0.397**	0.145
Tsum_4week	− 0.116	− 0.285	− 0.139	0.201	− 0.147	− **0.762**	− 0.242	0.244
Pressure_difference	− **0.105**	− 0.064	− 0.099	− **0.134**	0.042	− 0.118	− **0.171**	− **0.173**
Psum_4week	0.08	0.099	− 0.056	0.159	0.091	− 0.068	0.047	**0.394**
RH_3day	0.068	**0.372**	0.062	− 0.326	0.159	0.416	**0.202**	− 0.092
RH_1week	− 0.065	0.213	− 0.263	− 0.466	− **0.168**	0.411	0.092	− 0.15
RH_2week	− 0.068	0.173	− **0.269**	− **0.485**	− 0.14	0.429	0.106	− 0.235
RH_3week	− 0.014	0.229	− 0.266	− 0.439	− 0.025	**0.506**	0.145	− 0.2

### Collinearity diagnostics

**Figure 15:**
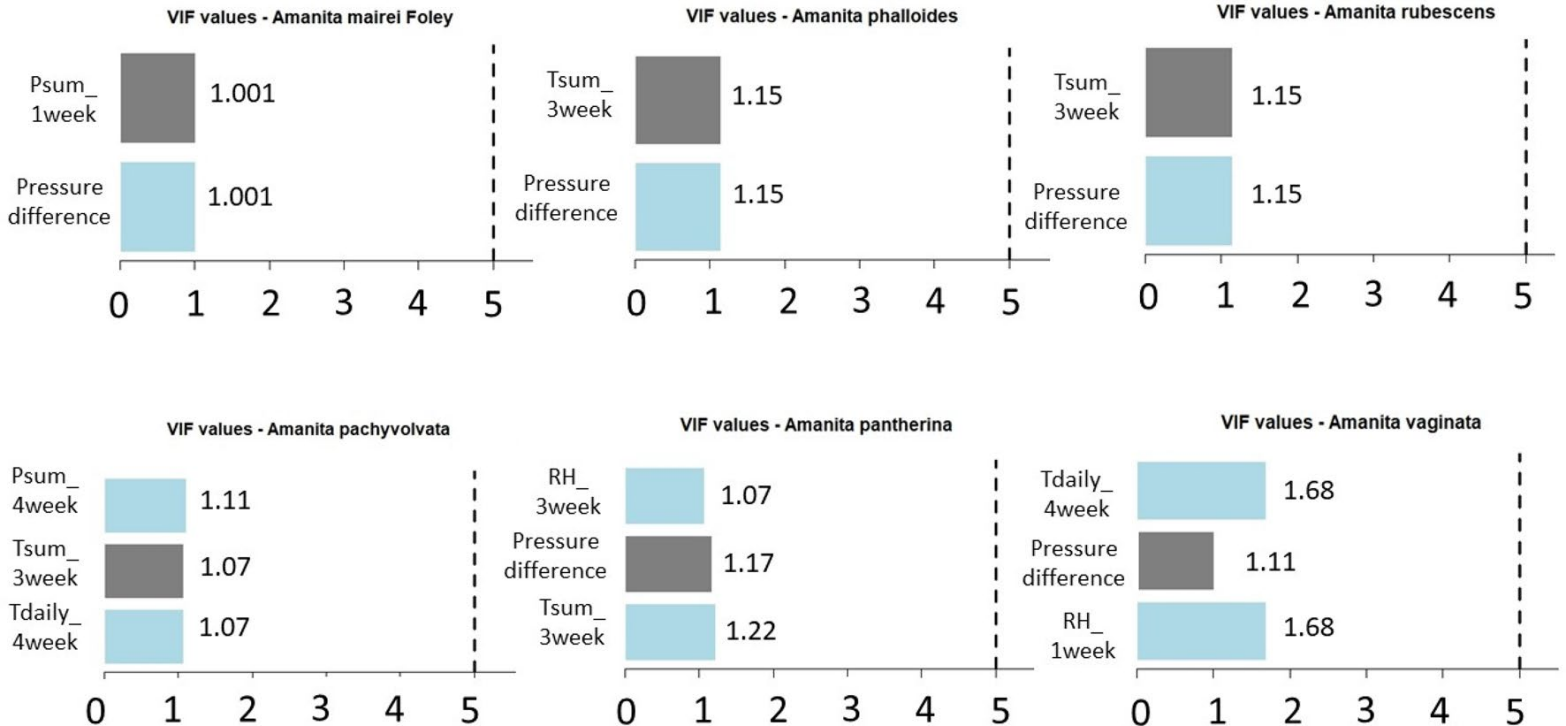
VIF values based on the predictor variables which were used to estimate the fruitbody formation of Amanitas in ANN.

**Figure 16:**
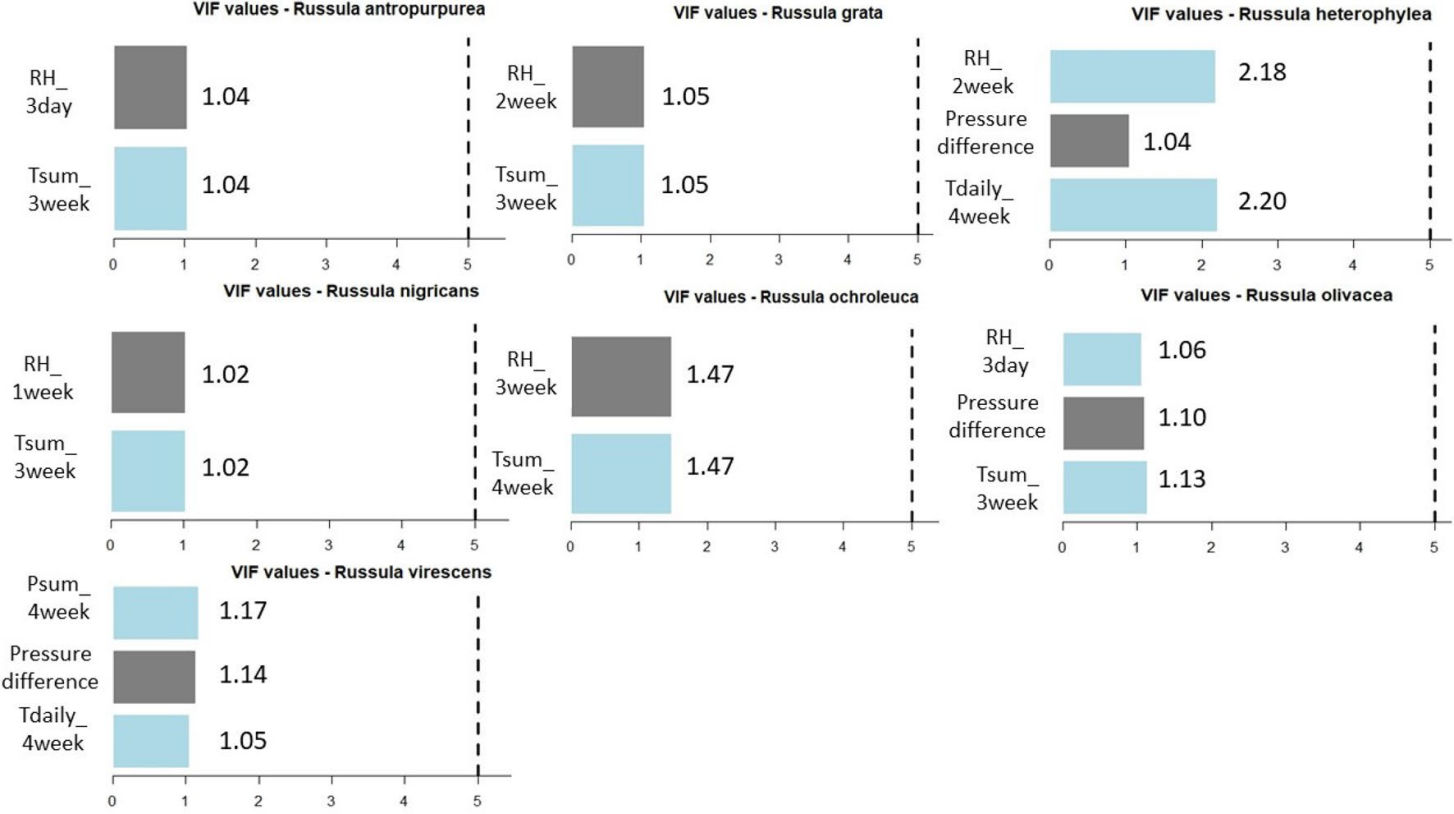
VIF values based on the predictor variables which were used to estimate the fruitbody formation of Russulas in ANN.

**Figure 17:**
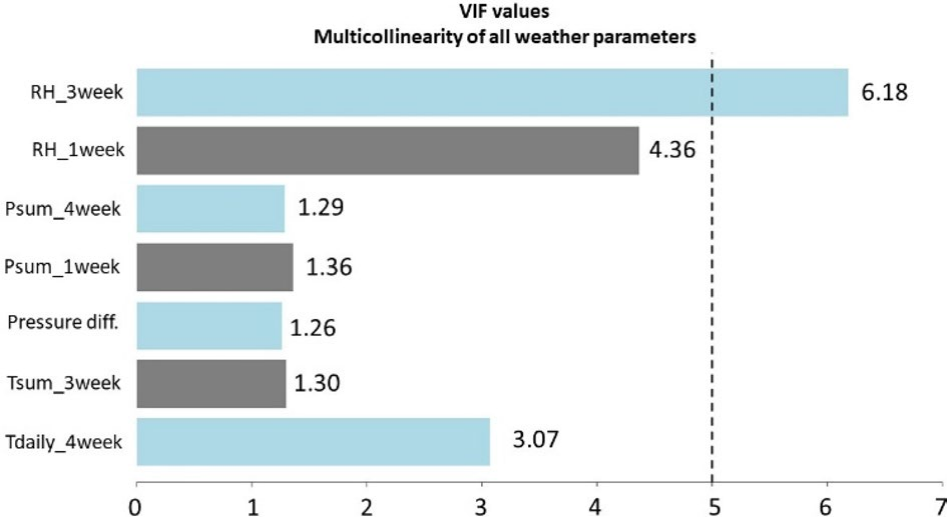
VIF values, where all the weather variables seen in Fig. 15 were included in the multicollinearity calculations for Amanita species in order to show that involving all the weather parameters would not lead to an appropriate result.

**Figure 18:**
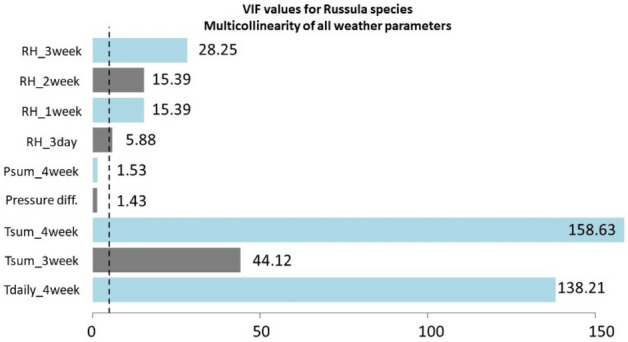
VIF values, where all the weather variables seen in Fig. 16 were included in the multicollinearity calculations for Russula species in order to show that involving all the weather parameters would not lead to an appropriate result.

The High Variance Inflation Factor (VIF) was used to check the collinearity. First, the weather parameters belonging to the ANN calculations of each *Amanita* and *Russula* species were checked (Figs. 15 and 16), and then all the weather parameters were used in order to show that involving all the weather parameters would not lead to an appropriate result (Figs. 17, 18).

The values of VIF range from 1 to infinity. A general rule of thumb for interpreting VIF is as follows^79^:

- VIF ≈ 1 there is no correlation between a given predictor variable and any other predictor variables in the model.
- VIF ϵ (1, 5] moderate correlation between the predictor variable and other predictor variables in the model, however, the severity is typically not significant enough to necessitate immediate attention.
- VIF > 5 potentially strong correlation between the predictor variable and other predictor variables in the model. Under these circumstances, the coefficient estimates and p-values presented in the regression output are likely to be unreliable.

It can be stated that there is no collinearity among the independent variables in any case of the ANN predictions of fruitbody production regarding to the *Amanita* species (Fig. 15) and *Russula* species (Fig. 16).

### Artificial neural network

Most processes in ecosystems behave non-linearly, therefore their mathematical approach should be able to satisfy that condition. Recently, the widely used learning algorithms, such as artificial neural networks, are able to handle and solve this scientific issue effectively.

**Figure 19:**
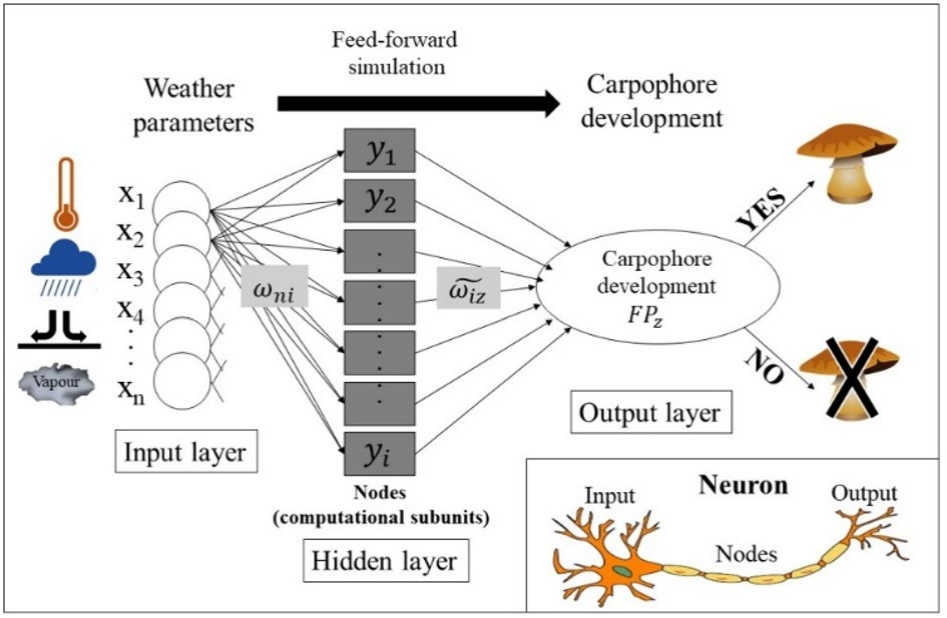
Sematic model of an artificial neural network. Based on the neurons’ connections in the brain, there are similarly interconnected groups of nodes in the model. The input layer contains the meteorological parameters as independent variables, and the output layer obtains the binomially distributed (Yes/No), dependent values of fungal sporocarp production as the result of non-linear calculations. The hidden layer is a so-called black box, where the minimization of errors is computed by a learning mechanism using the training dataset.

In this study, multilayer perceptrons (MLP) were used with backpropagation algorithm for training feed-forward neural networks^9^. In an ANN model, similarly to the neurons’ connection in the brain, there are interconnected groups of nodes. Our model consists of three layers (Fig. 19). The input layer contains the meteorological parameters as independent variables. The hidden layer, which can be seen as a black box, obtains the weights by calculating the gradient of the loss function and tries to minimize the errors by iterating always backwards from the last layer. These computational subunits in the hidden layer are called nodes. The output layer obtains the binomially distributed (Yes/No) dependent values of fruitbody formation as the result of non-linear calculations. Our ANN model is supervised, i.e. the training session learns from the known values of meteorological input data and the fungal output dataset. The testing session of the ANN model applies the mathematical formula and the weights obtained from the training session and estimates the output of fungal occurrences (Yes/No). The formula for calculating the *i*th node (y_i_) in the hidden layer (Fig. 19) is the following:

1 $${y}_{i}=\left(\sum_{j=1}^{n}{w}_{ji}{x}_{j}+{b}_{i}\right),$$

where “i” refers to the number of the neuron in the hidden layer, w_j i_ refers to the weight calculated from the nth meteorological parameter in the input layer, and b_i_ means the bias term for the i^th^ node. The activation function was a sigmoid function, i.e.

2 $$f\left(x\right)=\frac{1}{1+{e}^{-x}},$$

As a chain, the output layer (fruitbody production, FP) can be calculated by using y_i,_ as a new input, e.g.

3 $${FP}_{z}=\sum_{i}\widetilde{{w}_{iz}}{y}_{i}+\widetilde{{b}_{z}},$$

During the optimization process, the ANN is trained to minimize the cost function between the estimated and observed data of fungal production by finding the optimal values of ${w}_{ji, }{b}_{i}, \widetilde{{w}_{iz},}\widetilde{{b}_{z}}$^75^. The rate of training and test sessions in the dataset is 70% and 30%, respectively^80^. The order of the data was shuffled randomly in every calculation so that the 70%-30% split did not affect the results. The number of iterations in each ANN calculation was 100, from which the version with the lowest error was chosen as the best estimation of fruitbody development.

The possible source of error is the so-called overfitting^81^. This means that the model has more connections and weights than data in the input dataset. In our case, the input dataset in every case is more times bigger than the number of connections (see Figs. 1 and 14).

The ANN calculations were performed with R v4.1.2 statistical software^82^ using the R package „neuralnet” library^83^.

#### Alternative ANN methods

In this study, we had to find a method suitable for estimating binary outputs (fungal fruitbody formation YES/NO) based on continuous input data (meteorological parameters), and this method also had to be able to handle non-linear ecological systems. It has long been known that artificial neural networks are more successful than traditional regression methods^84^. In addition, several studies have shown that neural networks are suitable for binary classification. Jeatrakul and Wong^85^ tested five **artificial neural network** methods for binary outputs, namely back propagation neural network (BPNN), radial basis function neural network (RBFNN), general regression neural network (GRNN), probabilistic neural network (PNN), and complementary neural network (CMTNN). **Radial basis function neural network** (RBFNN) is similar to BPNN (which is used in our study), the only difference is in the training speed^86,87^. In our case, the training speed is out of consideration as the dataset did not require such troubleshooting. As for the **general regression neural network**, according to Specht^88^, the GRNN is more suitable for estimating continuous output data. Rutkowski^89^ also highlighted the effectiveness of GRNN based on a non-parametric regression method for the identification of time-varying continuous outputs. As a consequence, it was considered more appropriate to choose another method in our study. The **probabilistic neural network** is a type of radial basis network^90^. In the cases of probabilistic neural networks, the sigmoid activation function is replaced by an exponential function. The computational time can be drastically reduced, so this technique may be a good solution for problems in which the incremental adaptation time of back-propagation is a significant fraction of the total computation time^91^ (Specht, 1990). So that was out of the scope in our case. At last, the **complimentary neural network** must be taken into consideration as an alternative method. CMTNN uses two opposite feedforward backpropagation neural networks, the so-called Truth and Falsity Neural Networks. However, the literature has a poor description of the method, Jeatrakul and Wong^85^ found that the best method for binary classification problems is CMTNNs. Based on the above-introduced neural network methods, the back-propagation neural network (BPNN) seemed to be the best solution to estimate fungal fruitbody formation as a binary classification problem in our study.

### Validation of results

The validation of the prediction of the fruitbody formation was achieved based on the contingency table shown in Table 3. following Fagerland et al.^92^. The verification indices are seen in Table 3. The five most relevant verification indices used in practice are Accuracy (ACC), Bias (B), Probability of detection (POD), False alarm ratio (FAR) and Critical success index (CSI). All of the indices are dimensionless. ACC, POD, FAR and CSI varies between 0 and 1, while B shows that the method overforecasts or underforecasts the event (Table 4).

**Table 3:** Contingency table for the verification of ANN models.

		Observed		
		Fruitbody	No Fruitbody	
Forecast	Fruitbody	a = hit	b = false alarm	a + b = Forecast Fruitbody
	No Fruitbody	c = missed	d = correct negative	c + d = Forecast no Fruitbody
		a + c = Observed Fruitbody	b + d = Observed no Fruitbody

**Table 4:** Verification indices based on the terms in Table 3.

Index	Formula	Assessment
Accuracy	$ACC= \frac{a+d}{a+b+c+d}$	ACC ϵ [0; 1] ACC = 1 for a perfect forecast
Bias	$B= \frac{a+b}{a+c}$	B > 1, the event is overforecast, B < 1, the event is underforecast
Probability of detection	$POD= \frac{a}{a+c}$	POD ϵ [0; 1] POD = 1 for a perfect forecast
False alarm ratio	$FAR= \frac{b}{a+b}$	FAR ϵ [0; 1] FAR = 0 for a perfect forecast
Critical success index	$CSI= \frac{a}{a+b+c}$	CSI ϵ [0; 1] CSI = 1 for a perfect forecast

### Declaration

Experimental research and field studies on plants comply with relevant institutional, national, and international guidelines and legislation.

### Supplementary Information

Supplementary Information 1.

Supplementary Information 2.

Supplementary Information 3.

Supplementary Information 4.

Supplementary Information 5.

Supplementary Information 6.

## Data Availability

The datasets analysed during the current study are not publicly available due to private proprietary but are available from the corresponding author on reasonable request.

## References

[1] Sakamoto Y (2018). Influences of environmental factors on fruiting body induction, development and maturation in mushroom-forming fungi. Fungal Biol. Rev..

[2] Boddy L, Büntgen U, Egli S, Gange AC, Heegaard E, Kirk PM, Kauserud H (2014). Climate variation effects on fungal fruiting. Fungal Ecol..

[3] Vogt-Schilb H, Richard F, Malaval JC, Rapior S, Fons F, Bourgade V, Moreau PA (2022). Climate-induced long-term changes in the phenology of Mediterranean fungi. Fungal Ecol..

[4] Busch S, Braus GH (2007). How to build a fungal fruit body: From uniform cells to specialized tissue. Mol. Microbiol..

[5] Cao Y, Wu G, Yu D (2021). Include macrofungi in biodiversity targets. Science.

[6] Bonet JA, Pukkala T, Fischer CR, Palahí M, Aragón JM, Colinas C (2008). Empirical models for predicting the production of wild mushrooms in Scots pine (*Pinus sylvestris* L.) forests in the Central Pyrenees. Ann. For. Sci..

[7] Talley SM, Coley PD, Kursar TA (2002). The effects of weather on fungal abundance and richness among 25 communities in the Intermountain West. BMC Ecology.

[8] Krebs CJ, Carrier P, Boutin S, Boonstra R, Hofer E (2008). Mushroom crops in relation to weather in the southwestern Yukon. Botany.

[9] Taye ZM, Martínez-Peña F, Bonet JA, Martínez de Aragón J, de-Miguel S,  (2016). Meteorological conditions and site characteristics driving edible mushroom production in Pinus pinaster forests of Central Spain. Fungal Ecology.

[10] Laganà A, Angiolini C, Loppi S, Salerni E, Perini C, Barluzzi C, De Dominicis V (2002). Periodicity, fluctuations and successions of macrofungi in fir forests (Abies alba Miller) in Tuscany, Italy. For. Ecol. Manag..

[11] Liu Z, Peng C, Work T, Candau JN, DesRochers A, Kneeshaw D (2018). Application of machine-learning methods in forest ecology: Recent progress and future challenges. Environ. Rev..

[12] Arnolds E (1995) Problems in measurements of species diversity of macrofungi. In: Microbial diversity and ecosystem function (eds.: Allsopp D., Colwell R. R., Hawksworth D. L.). *CAB International, pp.* 337–353

[13] Liu D, Li J, Xiao N (2018). Survey methods and indicator system of assessment for macrofungal diversity in China. J. Nanjing For. Univ..

[14] Arnolds, E. Problems in measurements of species diversity of macrofungi. In: Microbial diversity and ecosystem function (eds.: Allsopp D., Colwell R. R., Hawksworth D. L.). *CAB International, pp.* 337–353 (1995).

[15] Runge A (1986). Pilzsukzession auf Kiefernstümpfen II. Zeitschrift für Mykologie.

[16] Runge A (1989). Elfjährige Pilzkundliche Untersuchungen im nordöstlichen Sauerland. Zeitschrift für Mykologie.

[17] Krisai-Greilhuber I (1992). Die Makromyceten im Raum von Wien.

[18] Rimóczi I, Pál-Fám F, Siller I, Jakucs E, Vasas G (2000) Proposal for the elaboration of the "Macrofungi" component of the National Biodiversity Monitoring System (third revision) /In Hungarian/. Submitted to the Authority for Nature Conservation.

[19] Senn-Irlet B (1987) Macromycetes as an element of forest structure in the region of Bern. In: Studies on Fungal communities (ed.: Pacioni, G.). *Soc. Bot. Italiana, L’Aquila*, pp. 195–219.

[20] Arnolds E (1992) The analysis and classification of fungal communities with special reference to macrofungi. In: Fungi in vegetation science (ed.: Winterhoff W.). *Kluwer Academic Publishers*, Dordrecht, Boston, London, pp. 7–47. 10.1007/978-94-011-2414-0_2

[21] Murakami Y (1987). Spatial distribution of Russula species in Castanopsis cuspidata forest. Trans. Br. Mycol. Soc..

[22] Pál-Fám F (2001). Review of methods used in macrofungal coenology /In Hungarian/. Botanikai Közlemények.

[23] Osono T (2015). Diversity, resource utilization, and phenology of fruiting bodies of litter-decomposing macrofungi in subtropical, temperate, and subalpine forests. J. For. Res..

[24] Łuszczyński J, Adamska E, Wojciechowska A, Czerwik-Marcinkowska J (2022). Diversity Patterns of Macrofungi in Xerothermic Grasslands from the Nida Basin (Małopolska Upland, Southern Poland): A Case Study. Biology.

[25] McCulloch W, Pitts W (1943). A logical calculus of ideas immanent in nervous activity. Bull. Math. Biophys..

[26] Park YS, Chon TS, Kwak IS, Lek S (2004). Hierarchical community classification and assessment of aquatic ecosystems using artificial neural networks. Sci. Tot. Environ..

[27] Kattenborn T, Eichel J, Fassnacht FE (2019). Convolutional Neural Networks enable efficient, accurate and fine-grained segmentation of plant species and communities from high-resolution UAV imagery. Sci. Rep..

[28] Wolski GJ, Kruk A (2020). Determination of plant communities based on bryophytes: The combined use of Kohonen artificial neural network and indicator species analysis. Ecol. Indic..

[29] Zhang C, Chen Y, Xu B, Xue Y, Ren Y (2020). Improving prediction of rare species’ distribution from community data. Sci. Rep..

[30] Elizondo DA, McClendon RW, Hoogenboom G (1994). Neural network models for predicting flowering and physiological maturity of soybean. Trans. ASAE.

[31] Olden JD (2000). An artificial neural network approach for studying phytoplankton succession. Hydrobiologia.

[32] KüÇükönder H, Boyaci S, Akyüz A (2016). A modeling study with an artificial neural network: Developing estimationmodels for the tomato plant leaf area. Turk. J. Agric. For..

[33] Ghazvinei PT, Hassanpour DH, Mosavi A, Yusof K, bin W, Alizamir M, Shamshirband S, Chau K,  (2018). Sugarcane growth prediction based on meteorological parameters using extreme learning machine and artificial neural network. Eng. Appl. Comput. Fluid Mech..

[34] Supriyanto Noguchi R, Ahamed T, Rani DS, Sakurai K, Nasution MA, Watanabe MM (2018). Artificial neural networks model for estimating growth of polyculture microalgae in an open raceway pond. Biosyst. Eng..

[35] Coutinho FH, Thompson CC, Cabral AS, Paranhos R, Dutilh BE, Thompson FL (2019). Modelling the influence of environmental parameters over marine planktonic microbial communities using artificial neural networks. Sci. Total Environ..

[36] Thippa RG, Swarna Priya RM, Parimala M, Chowdhary CL, Praveen Kumar RM, Hakak S, Khan WZ (2020). A deep neural networks based model for uninterrupted marine environment monitoring. Comput. Commun..

[37] Hsieh WW, Tang B (1998). Applying neural network models to prediction and data analysis in meteorology and oceanography. Bull. Am. Meteorol. Soc..

[38] Sharma, P., Singh, B. K., & Singh, R. P. Prediction of potato late blight disease based upon weather parameters using artificial neural network approach. In *9th International Conference on Computing, Communication and Networking Technologies (ICCCNT)* (2018)*.*10.1109/icccnt.2018.8494024*.*

[39] Bayat M, Ghorbanpour M, Zare R, Jaafari A, Thai Pham B (2019). Application of artificial neural networks for predicting tree survival and mortality in the Hyrcanian forest of Iran. Comput. Electron. Agric..

[40] Pohan S, Warsito B, Suryono S (2020). Backpropagation artificial neural network for prediction plant seedling growth. J. Phys.: Conf. Ser..

[41] Lidasan JU, Tagacay MP (2018). Mushroom recognition using neural network. Int. J. Comput. Sci. Iss..

[42] Verma SK, Dutta M (2018). Mushroom classification using ANN and ANFIS algorithm. IOSR J. Eng..

[43] Preechasuk, J., Chaowalit, O., Pensiri, F., Visutsak, P. Image Analysis of Mushroom Types Classification by Convolution Neural Networks. In: *Proceedings of the 2019 2nd artificial intelligence and cloud computing conference* (2019). 10.1145/3375959.3375982

[44] Ardabili FS, Najafi B, Ghaebi H, Shamshirband S, Mostafaeipour A (2017). A novel enhanced exergy method in analyzing HVAC system using soft computing approaches: A case study on mushroom growing hall. J. Build. Eng..

[45] Ardabili, S., Mosavi, A., Mahmoudi, A., Gundoshmian, T. M., Nosratabadi, S., Várkonyi-Kóczi, A. R. Modelling temperature variation of mushroom growing hall using artifical neural networks. In: Várkonyi-Kóczy, A. R. (Ed.) *Engineering for Sustainable Future. Lecture Notes in Networks and Systems* 33–45. (Springer, Switzerland, 2020). 10.1007/978-3-030-36841-8

[46] Mohebbi M, Fathi M, Shahidi F (2010). Genetic algorithm-artificial neural network modeling of moisture and oil content of pretreated fried mushroom. Food Bioprocess Technol..

[47] Salvador C, Martins MR, Vicente H, Neves J, Arteiro JM, Caldeira AT (2012). Modelling molecular and inorganic data of Amanita ponderosa mushrooms using artificial neural networks. Agrofor. Syst..

[48] Baltacıoğlu H, Bayındırlı A, Severcan M, Severcan F (2015). Effect of thermal treatment on secondary structure and conformational change of mushroom polyphenol oxidase (PPO) as food quality related enzyme: A FTIR study. Food Chem..

[49] Omari A, Behroozi-Khazaei N, Sharifian F (2018). Drying kinetic and artificial neural network modeling of mushroom drying process in microwave-hot air dryer. J. Food Process Eng..

[50] Yin H, Yi W, Hu D (2022). Computer vision and machine learning applied in the mushroom industry: A critical review. Comput. Electron. Agric..

[51] Lee, C. H., Choi, D., Pecchia, J., He, L., & Heinemann, P. Development of a mushroom harvesting assistance system using computer vision. In *An ASABE Annual International Meeting*, 1900505 (2019). 10.13031/aim.201900505

[52] Heinemann PH, Hughes R, Morrow CT, Sommer HJ, Beelman RB, Wuest PJ (1994). Grading of mushrooms using a machine vision system. Trans. ASAE.

[53] Chen HH, Ting CH (2004). The development of a machine vision system for shiitake grading. J. Food Qual..

[54] Wang FY, Feng WJ, Zheng JY, Sun JB, Niu LY, Chen ZX, Zhang XT, Wang L (2018). Design and experiment of automatic sorting and grading system based on machine vision for white Agaricus bisporus. Trans. Chin. Soc. Agric. Eng..

[55] Baragatti M, Grollemund PM, Montpied P, Dupouey JL, Gravier J, Murat C, Le Tacon F (2019). Influence of annual climatic variations, climate changes, and sociological factors on the production of the Périgord black truffle (Tuber melanosporum Vittad.) from 1903–1904 to 1988–1989 in the Vaucluse (France). Mycorrhiza.

[56] Steidinger BS, Büntgen U, Stobbe U, Tegel W, Sproll L, Haeni M, Peter M (2022). The fall of the summer truffle: Recurring hot, dry summers result in declining fruitbody production of Tuber aestivum in Central Europe. Glob. Change Biol..

[57] Sun JW, Zhao KX, Ji JT, Zhu XF, Ma H (2021). Detection and diameter measurement method of agaricus bisporus based on “Submerged Method. J. Agric. Mech. Res..

[58] Zhou J, Ding WJ, Zhu XJ, Niu XM (2017) Evaluation on formation rate of Pleurotus eryngii primordium under different humidity conditions by computer vision. *J. Zhejiang Univ. (Agric. Life Sci.)* **43(2)**, 262–272. 10.3785/j.issn.1008-9209.2016.04.113

[59] Alkronz ES, Moghayer KA, Meimeh M, Gazzaz M (2019). Classification of mushroom using artificial neural network. Int. J. Acad. Appl. Res..

[60] Aljojo MS, Dawood KJ, Zaqout MH, Salem RM (2021). ANN for mushroom prediction. Int. J. Acad. Multidiscip. Res..

[61] Adhitya, R. Y., Ramadhan, M. A., Kautsar, S., Rinanto, N., Sarena ST, Munadhif I, Soeprijanto A (2016) Comparison methods of Fuzzy Logic Control and Feed Forward Neural Network in automatic operating temperature and humidity control system (Oyster Mushroom Farm House) using microcontroller. *Paper presented at the International Symposium on Electronics and Smart Devices (ISESD)*, Bandung, Indonesia, 29–30 Nov 2016. 10.1109/isesd.2016.7886713

[62] Hernández-Rodríguez M, de-Miguel S, Pukkala T, Oria-de-Rueda JA, Martín-Pinto P,  (2015). Climate-sensitive models for mushroom yields and diversity in Cistus ladanifer scrublands. Agric. For. Meteorol..

[63] Solanki DS, Kumar S, Sharma K, Gehlot P, Singh SK (2016). Weather prerequisites for fructification of *Phellorinia* mushroom. Plant Arch..

[64] Primicia I, Camarero JJ, Martínez de Aragón J, de-Miguel S, Bonet JA,  (2016). Linkages between climate, seasonal wood formation and mycorrhizal mushroom yields. Agric. For. Meteorol..

[65] Liu YS, Liu J, Kumla J, Lumyong S (2022). Two New *Amanita* Species in Section *Amanita* from Thailand. Diversity.

[66] Cui YY, Cai Q, Yang ZL (2021). *Amanita chuformis*, a new *Amanita* species with a marginate basal bulb. Mycoscience.

[67] Cui YY, Cai Q, Tang LP, Liu JW, Yang ZL (2018). The family *Amanitaceae*: molecular phylogeny, higher-rank taxonomy and the species in China. Fungal Divers..

[68] Vizzini A, Cingarlini C, Sartori D, Maraia GL, Setti L, Poumarat S, Kudzma L, Dovana F (2020). Assessing the taxonomic status of Amanita citrina var. intermedia (Basidiomycota, Agaricales). Phytotaxa.

[69] Alvarado P, Gasch-Illescas A, Morel S, Dagher-Kharrat MB, Moreno G, Manjón JL, Carteret X, Bellanger JM, Rapor S, Gelardi M, Moreau PA (2022). *Amanita* section *Phalloideae* species in the mediterranean basin: Destroying angels reviewed. Biology.

[70] Siller I, Dima B, Albert L, Vasas G, Fodor L, Pál-Fám F, Bratek Z, Zagyva I (2006). Protected Macrofungi in Hungary. Clusiana.

[71] Siller I, Kutszegi G, Takács K, Varga T, Zs M, Turcsányi G, Ódor P, Dima B (2013). Sixty-one macrofungi species new to Hungary in Őrség National Park. Mycosphere.

[72] Kutszegi G, Siller I, Dima B, Zs M, Varga T, Takács K, Turcsányi G, Bidló A, Ódor P (2021). Revealing hidden drivers of macrofungal species richness by analyzing fungal guilds in temperate forests, West Hungary. Community Ecol..

[73] Kovácsné, L. E., & Török, K. Nemzeti Biodiverzitás-monitorozó (1997).

[74] Rendszer III. Növénytársulások, társuláskomplexek és élőhelymozaikok. Hungarian Natural History Museum, Budapest, ISBN 963 7093 46 X

[75] De-Wei LI (2005). Release and dispersal of basidiospores from Amanita muscaria var. alba and their infiltration into a residence. Mycol. Res..

[76] Haykin, S. Neural Networks and Learning Machines. Vol. 3. (Pearson Upper Saddle River, NJ, 2009).

[77] Szentimrey T, Bihari Z (2004) Mathematical background of the spatial interpolation methods and the software MISH (Meteorological Interpolation based on Surface Homogenized Data Basis). In *Proceedings of Spatial Interpolation in climatology and meteorology, COST Action 719, The Use of Geographic Information Systems in Climatology and Meteorology*. Budapest, Hungary, ISBN: 92-898-0033-X

[78] Peel MC, Finlayson BL, McMahon TA (2007). Updated world map of the Köppen–Geiger climate classification. Hydrol. Earth Syst. Sci..

[79] Boslaugh A, Watters PA (2008) *Statistics in a Nutshell: A Desktop Quick Reference*. O'Reilly Media, Sebastopol, Canada, ISBN-**13**, 978-0596510497

[80] Stine RA (1995). Graphical interpretation of variance inflation factors. Am. Stat..

[81] Gholami V, Torkaman J, Dalir P (2019). Simulation of precipitation time series using tree-rings, earlywood vessel features, and artificial neural network. Theor. Appl. Climatol..

[82] Livingstone DJ, Manallack DT, Tetko IV (1997). Data modelling with neural networks: advantages and limitations. J. Comput. Aided Mol. Des..

[83] R Core Team. R: A language and environment for statistical computing. R Foundation for Statistical Computing. Vienna, Austria (2021). https://www.R-project.org/. Accessed 01 11 2021

[84] Fritsch, S., Guenther, F., Wright, M. N. Neuralnet: Training of Neura Networks. R package version 1.44.2 (2019). https://CRAN.R-project.org/package=neuralnet. Accessed 07 02, 2019

[85] Murtagh F (1991). Multilayer perceptrons for classification and regression. Neurocomputing.

[86] Jeatrakul P, Wong KW (2009). Comparing the performance of different neural networks for binary classification problems. Eighth International Symposium on Natural Language Processing, Bangkok, Thailand.

[87] Schilling RJ, Carroll JJ, Al-Ajlouni AF (2001). Approximation of nonlinear systems with radial basis function neural networks. IEEE Trans. Neural Netw..

[88] Wu Y, Wang H, Zhang B, Du KL (2012). Using radial basis function networks for function approximation and classification. Int. Schol. Res. Not..

[89] Specht DF (1991). A general regression neural network. IEEE Trans. Neural Netw..

[90] Rutkowski L (2004). Generalized Regression Neural Networks in a Time-Varying Environment. In: *New Soft Computing Techniques for System Modeling, Pattern Classification and Image Processing. Studies in Fuzziness and Soft Computing*, **143**, Springer, Berlin, Heidelberg. 10.1007/978-3-540-40046-2_5

[91] Ooi S-Y, Teoh ABJ, Ong T-S (2008). Compatibility of biometric strengthening with probabilistic neural network. In *Biometrics and Security Technologies*, 2008. ISBAST 2008. International Symposium on Biometrics and Security Technologies.

[92] Specht DF (1990). Probabilistic neural networks. Neural networks.

[93] Fagerland, M., Lydersen, S., & Laake, P. Statistical Analysis of Contingency Tables. Chapman and Hall/CRC, New York, e-ISBN: 9781315371116 (2017). 10.1201/9781315374116

