# Supplementary material for: Verification study on how macrofungal fruitbody formation can be predicted by artificial neural network
Source: Sci Rep. 2024 Jan 2;14:278. doi: 10.1038/s41598-023-50638-8 (PMC10761683; doi:10.1038/s41598-023-50638-8)

**SUPPLEMENTARY MATERIAL 1**

**Verification study on how macrofungal fruitbody formation can be predicted by artificial neural network**

Katalin Somfalvi-Tóth^*^, Ildikó Jócsák, Ferenc Pál-Fám

**Journal:** Scientific Reports

***Corresponding author**: Department of Agronomy, Institute of Agronomy, Hungarian University of Agriculture and Life Sciences, 40 Guba S. str., H-7400 Kaposvár, Hungary, [somfalvi-toth.katalin@uni-mate.hu](mailto:somfalvi-toth.katalin@uni-mate.hu)

ANN models with the nodes in the hidden layer and the weights for each *Amanita* species calculated with **genus-level meteorological parameters** (Tsum_3weeks, pressure difference, and mean relative humidity for 3 days based on Table 1). Blue lines represent the bias terms, just like the intercept in a linear model. The results of *A. mairei* Foley can be seen in the manuscript in Figure 1.


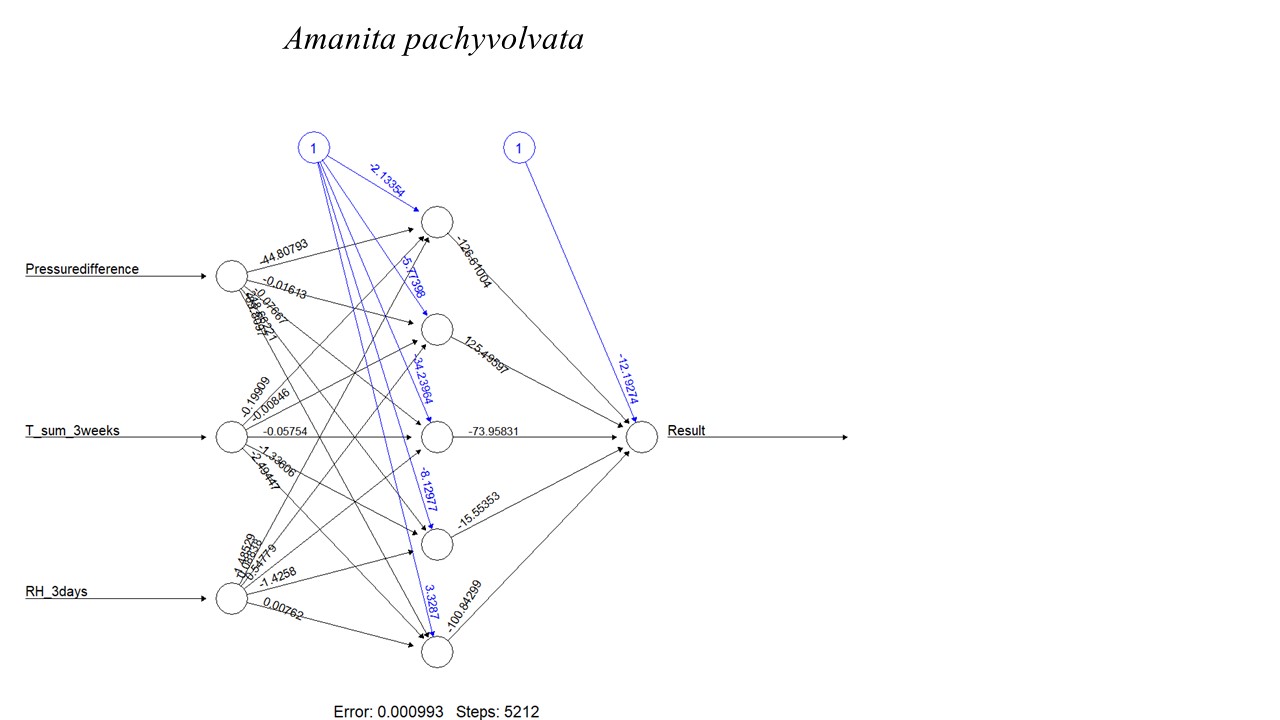


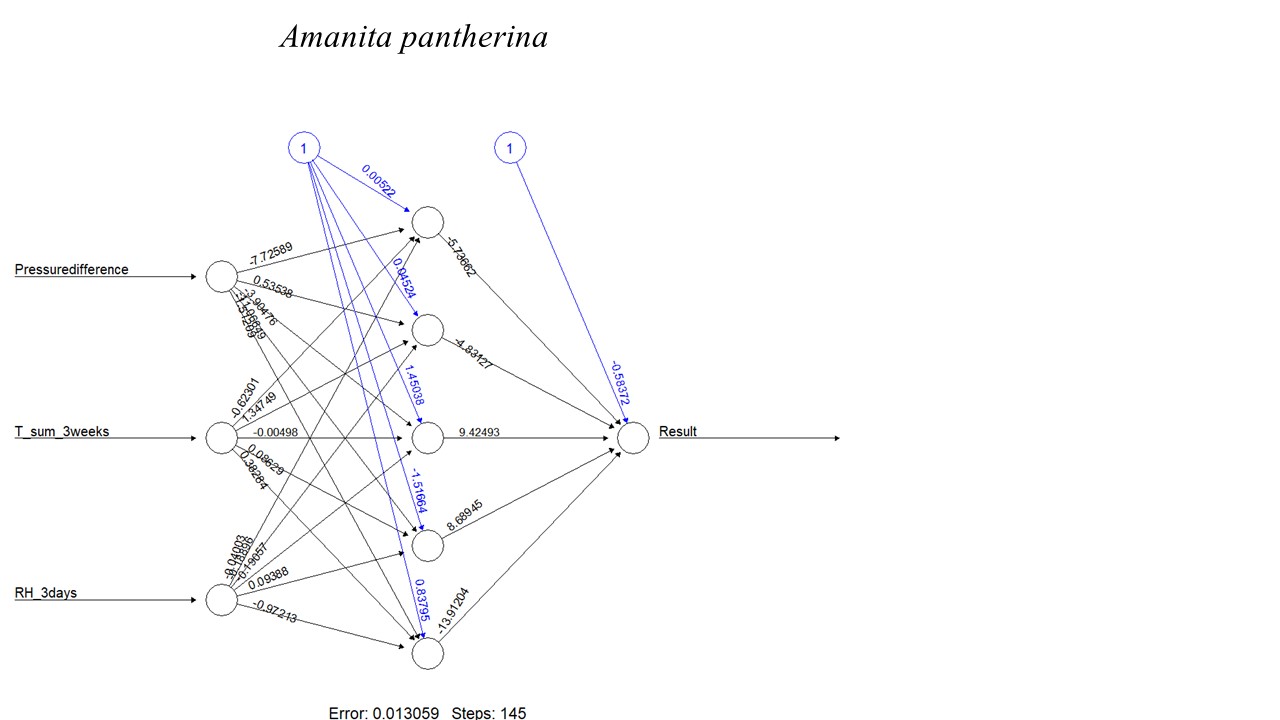

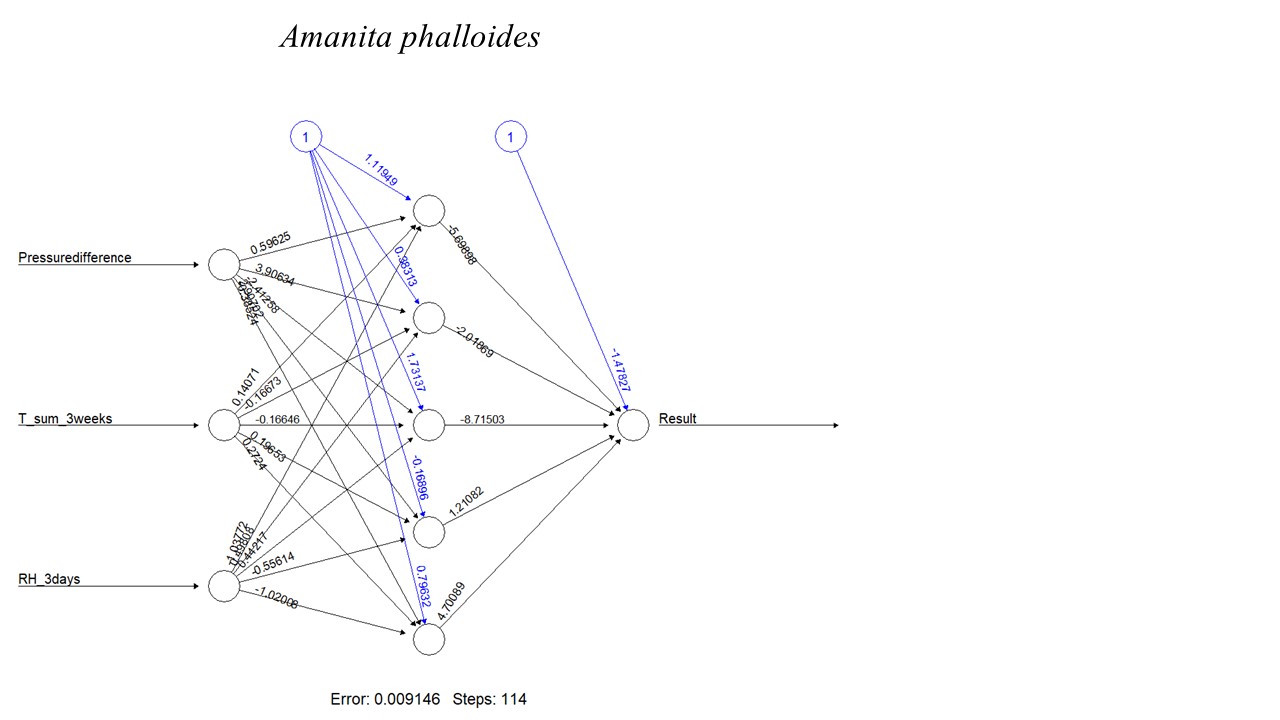

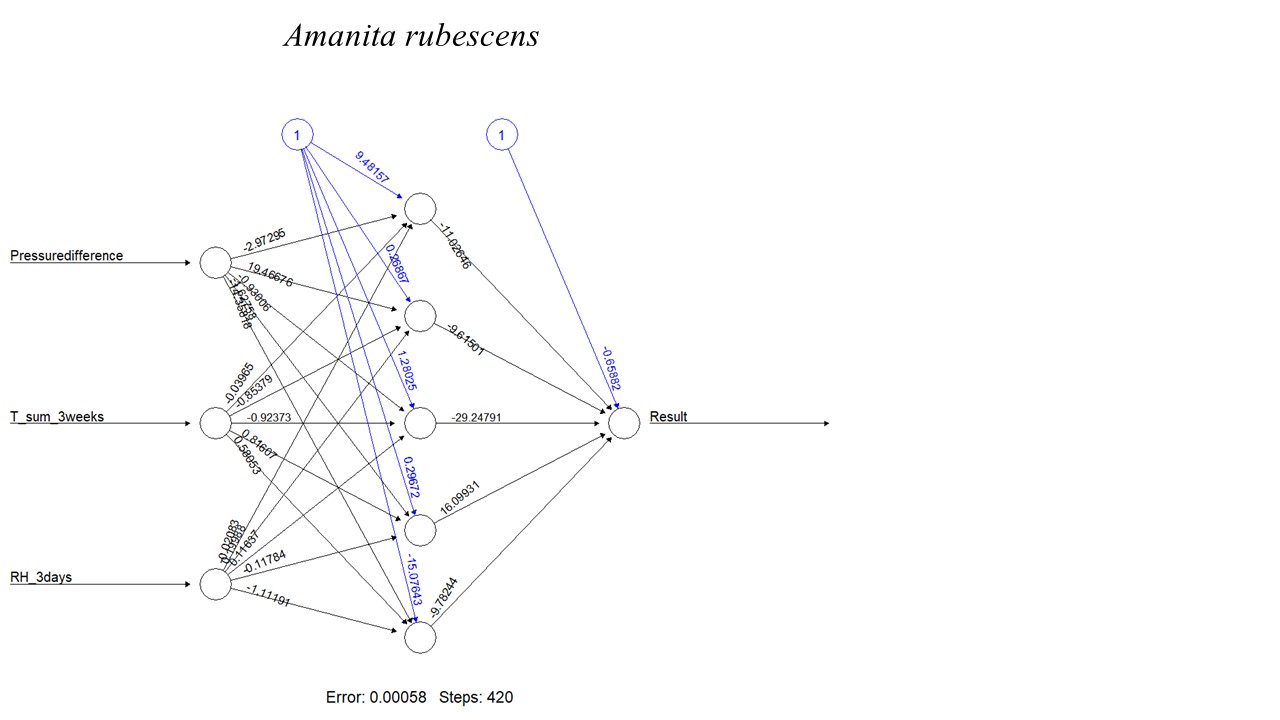

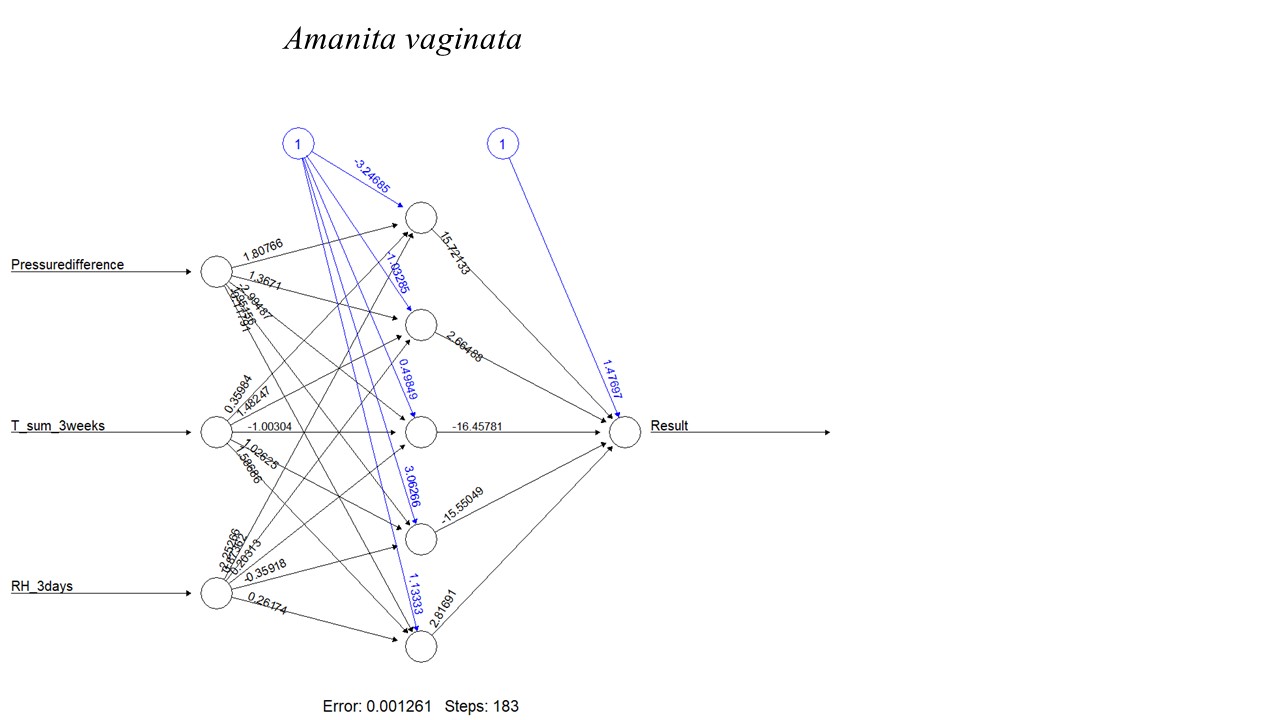

Supplement: Supplementary file 1 — Supplementary Information 1. [file 41598_2023_50638_MOESM1_ESM.docx]
