# Supplementary material for: Verification study on how macrofungal fruitbody formation can be predicted by artificial neural network
Source: Sci Rep. 2024 Jan 2;14:278. doi: 10.1038/s41598-023-50638-8 (PMC10761683; doi:10.1038/s41598-023-50638-8)
Supplement: Supplementary file 5 — Supplementary Information 5. [file 41598_2023_50638_MOESM5_ESM.docx]

**SUPPLEMENTARY MATERIAL 5**

**Verification study on how macrofungal fruitbody formation can be predicted by artificial neural network**

Katalin Somfalvi-Tóth^*^, Ildikó Jócsák, Ferenc Pál-Fám

Journal: Scientific Reports

*Corresponding author: Department of Agronomy, Institute of Agronomy, Hungarian University of Agriculture and Life Sciences, 40 Guba S. str., H-7400 Kaposvár, Hungary, [somfalvi-toth.katalin@uni-mate.hu](mailto:somfalvi-toth.katalin@uni-mate.hu)

*Table 7: Correlation table between occurence of Russula species and different meteorological variables in order to determine the most relevant meteorological parameters used as inputs for ANN models.* ***Fixed meteorology*** *means that the same weather parameters were applied for all Russulas in ANN calculations, so it was assumed that all Russula species have the same initial condition for growing.* ***Species-specific meteorology*** *means that selected meteorological parameters were applied for the calculation of ANNs, so the two or three strongest relationships (R^2^) were used for each Russula species, so it was assumed that each Russula species has its own initial condition for growing*. *The colored cells show the selected variables for ANN calculations.*

|  | **Fixed meteorology** | **Species-specific meteorology** | | | | | | |
| --- | --- | --- | --- | --- | --- | --- | --- | --- |
| **R^2^** | All *Russula* species | *R. antropurpurea* | *R. grata* | *R. heterophylea* | *R. nigricans* | *R. ochroleuca* | *R. olivacea* | *R. virescens* |
| Tdaily_1week | 0.036 | -0.051 | 0.099 | 0.425 | 0.138 | -0.562 | -0.136 | 0.265 |
| Tdaily_2week | 0.061 | -0.032 | 0.172 | 0.444 | 0.155 | -0.590 | -0.072 | 0.312 |
| Tdaily_3week | 0.063 | -0.032 | 0.225 | 0.451 | 0.149 | -0.592 | -0.038 | 0.340 |
| Tdaily_4week | 0.073 | -0.002 | 0.257 | 0.464 | 0.191 | -0.592 | -0.051 | 0.343 |
| Tsum_1week | 0.036 | -0.051 | 0.099 | 0.425 | 0.138 | -0.562 | -0.136 | 0.265 |
| Tsum_2week | 0.061 | -0.032 | 0.172 | 0.444 | 0.155 | -0.590 | -0.072 | 0.312 |
| Tsum_3week | 0.351 | 0.576 | 0.708 | 0.415 | 0.594 | 0.397 | 0.397 | 0.145 |
| Tsum_4week | -0.116 | -0.285 | -0.139 | 0.201 | -0.147 | -0.762 | -0.242 | 0.244 |
| Pressure_difference | -0.105 | -0.064 | -0.099 | -0.134 | 0.042 | -0.118 | -0.171 | -0.173 |
| Psum_1week | -0.034 | -0.034 | 0.214 | -0.110 | -0.037 | -0.136 | 0.156 | 0.113 |
| Psum_2week | -0.086 | -0.181 | 0.235 | -0.144 | -0.107 | -0.117 | 0.050 | 0.031 |
| Psum_3week | 0.017 | -0.007 | 0.049 | 0.048 | 0.144 | -0.132 | -0.019 | 0.243 |
| Psum_4week | 0.080 | 0.099 | -0.056 | 0.159 | 0.091 | -0.068 | 0.047 | 0.394 |
| RH_1day | 0.050 | 0.283 | -0.023 | -0.276 | 0.068 | 0.383 | 0.099 | -0.075 |
| RH_3day | 0.068 | 0.372 | 0.062 | -0.326 | 0.159 | 0.416 | 0.202 | -0.092 |
| RH_1week | -0.065 | 0.213 | -0.263 | -0.466 | -0.168 | 0.411 | 0.092 | -0.150 |
| RH_2week | -0.068 | 0.173 | -0.269 | -0.485 | -0.140 | 0.429 | 0.106 | -0.235 |
| RH_3week | -0.014 | 0.229 | -0.266 | -0.439 | -0.025 | 0.506 | 0.145 | -0.200 |
| RH_4week | -0.021 | 0.201 | -0.270 | -0.456 | -0.039 | 0.488 | 0.157 | -0.170 |
