# Supplementary material for: Verification study on how macrofungal fruitbody formation can be predicted by artificial neural network
Source: Sci Rep. 2024 Jan 2;14:278. doi: 10.1038/s41598-023-50638-8 (PMC10761683; doi:10.1038/s41598-023-50638-8)
Supplement: Supplementary file 6 — Supplementary Information 6. [file 41598_2023_50638_MOESM6_ESM.docx]

**SUPPLEMENTARY MATERIAL 6.**

**Verification study on how macrofungal fruitbody formation can be predicted by artificial neural network**

Katalin Somfalvi-Tóth^*^, Ildikó Jócsák, Ferenc Pál-Fám

Journal: Scientific Reports

*Corresponding author: Department of Agronomy, Institute of Agronomy, Hungarian University of Agriculture and Life Sciences, 40 Guba S. str., H-7400 Kaposvár, Hungary, [somfalvi-toth.katalin@uni-mate.hu](mailto:somfalvi-toth.katalin@uni-mate.hu)

*Table 8: Correlation table between occurence of Amanita species and different meteorological variables in order to determine the most relevant meteorological parameters used as inputs for ANN models.* ***Fixed meteorology*** *means that the same weather parameters were applied for all Amanitas in ANN calculations. so it was assumed that all Amanita species have the same initial condition for growing.* ***Species-specific meteorology*** *means that selected meteorological parameters were applied for the calculation of ANNs. so the two or three strongest relationships (R^2^) were used for each Amanita species. so it was assumed that each Amanita species has its own initial condition for growing. The colored cells show the selected variables for ANN calculations.*

|  | Fixed meteorology | Species-specific meteorology | | | | | |
| --- | --- | --- | --- | --- | --- | --- | --- |
| R^2^ | All *Amanita* species | *Amanita mairei Foley* | *Amanita pachyvolvata (Bon) Krieglst.* | *Amanita pantherina (DC.:Fr.) Krombh.* | *Amanita phalloides (Vaill. ex Fr.) Link* | *Amanita rubescens (Pers.:Fr.)Gray* | *Amanita vaginata (Bull.:Fr.)Vitt.* |
| Tdaily_1week | -0.038 | -0.046 | -0.003 | -0.209 | -0.013 | 0.167 | 0.199 |
| Tdaily_2week | 0.022 | 0.050 | 0.103 | -0.236 | 0.086 | 0.176 | 0.297 |
| Tdaily_3week | 0.032 | 0.061 | 0.175 | -0.211 | 0.087 | 0.136 | 0.293 |
| Tdaily_4week | 0.032 | 0.045 | 0.210 | -0.235 | 0.064 | 0.129 | 0.319 |
| Tsum_1week | -0.038 | -0.046 | -0.003 | -0.208 | -0.013 | 0.167 | 0.199 |
| Tsum_2week | 0.022 | 0.050 | 0.103 | -0.236 | 0.086 | 0.176 | 0.297 |
| Tsum_3week | 0.224 | 0.277 | 0.143 | -0.355 | 0.590 | 0.654 | 0.075 |
| Tsum_4week | -0.074 | -0.076 | 0.123 | -0.037 | -0.201 | -0.231 | 0.259 |
| Pressure_difference | 0.199 | 0.607 | 0.093 | -0.290 | 0.294 | 0.359 | 0.293 |
| Psum_1week | 0.089 | 0.319 | 0.158 | -0.018 | 0.191 | 0.144 | -0.065 |
| Psum_2week | 0.047 | 0.144 | 0.296 | -0.026 | 0.073 | -0.057 | -0.123 |
| Psum_3week | 0.056 | -0.061 | 0.395 | -0.043 | -0.104 | 0.122 | 0.022 |
| Psum_4week | 0.097 | 0.077 | 0.424 | -0.128 | 0.028 | 0.091 | 0.027 |
| RH_1day | 0.114 | 0.119 | 0.093 | 0.048 | 0.254 | 0.074 | -0.072 |
| RH_3day | 0.117 | 0.249 | 0.134 | 0.060 | 0.226 | 0.201 | -0.210 |
| RH_1week | -0.013 | 0.041 | -0.086 | 0.187 | 0.033 | -0.073 | -0.357 |
| RH_2week | 0.001 | -0.002 | -0.103 | 0.300 | 0.020 | -0.110 | -0.307 |
| RH_3week | 0.039 | -0.012 | -0.080 | 0.355 | 0.016 | 0.010 | -0.253 |
| RH_4week | 0.048 | 0.030 | -0.057 | 0.354 | 0.051 | -0.010 | -0.285 |
